# Supplementary material for: Understanding Mental Health in Developmental Dyslexia: A Scoping Review
Source: Int J Environ Res Public Health. 2023 Jan 16;20(2):1653. doi: 10.3390/ijerph20021653 (PMC9864451; doi:10.3390/ijerph20021653)
Supplement: Supplementary file 1 [file ijerph-20-01653-s001.zip › ijerph-2053746-supplementary.pdf]

Table S1: Source selection tool

	YES	No
**Publication type**	• Empirical studies – case study, qualitative, quantitative, intervention studies.<br>• Reviews (meta-analysis, systematic, scoping)<br>• Peer reviewed – don't worry about this one at title/abstract screening<br>• Dissertation and thesis<br>• English language	• Editorials<br>• Opinion pieces<br>• Policy/position papers<br>• Book or article reviews<br>• Learning modules<br>• Study protocols<br>• Introductions to special editions
**Population**	• Children (0-18)<br>• Primary or secondary students<br>• Parents/teachers/others reporting on childhood reading difficulties/mental health<br>• Adults retrospectively reporting about childhood reading difficulties	• Adults<br>• Non-human (e.g., rat studies)<br>• College/Uni samples<br>• second language
**Concept one: reading difficulties**	• Children with dyslexia<br>• Children with Specific Learning Disorder (sub-type not specified)	• Studies were a reading difficulties group or an SLD are not mentioned in the title/abstract

	• Learning difficulties/literacy difficulties<br>• Word reading or reading fluency difficulties<br>• Poor readers, struggling readers, children with reading difficulties, reading disorder<br>• Neurodevelopmental disorders – is there a learning/reading difficulties group mentioned in the title/abstract? Then YES.<br>• Poor comprehending in addition to poor word reading difficulties<br>• Co-occuring ADHD, language difficulties, or additional learning difficulties are to be expected.	• Children who are only poor comprehenders<br>• Poor reading due to:<br>• Reading difficulties associated with: lack of educational opportunity (e.g. Head start schools), biomedical factors (e.g., brain injury, Fragile X, epilepsy), sensory issues (e.g., hearing loss); autism
	**YES**	**NO**
**Concept 2– internalising/externalising mental health concerns**	**YES**<br>• Internalising behaviour (emotional difficulties; anxiety, depression, somatic complaints)<br>• Externalising behaviour (behaviour difficulties;	**No – in the absence of internalising/externalising**<br>• School refusal<br>• Attentional difficulties<br>• Incarceration<br>• NSSI<br>• Suicidal behaviour

	(hyperactivity, impulsivity, conduct problems (e.g., aggression, rule-breaking)<br>• Externalising disorders (ADHD, CD, ODD).<br>• Internalising disorders (e.g., depression, anxiety disorder sub-types, reading/test anxiety)<br>• Psycho-social or socio-emotional difficulties assessed by global measures<br>• For qualitative studies parent, child, teacher report of mental health/emotional difficulties is sufficient for inclusion.	• Substance abuse<br>• Eating disorders<br>• Peer problems (e.g., bullying victimisation).<br>• Psychosis<br>• Self-esteem, self-concept, self-perception<br>• Stress
**Concept 3: Third factor**	• Qual studies – another factor other than internalising/externalisingexplored e.g., self-esteem, coping strategies.<br>• Individual level (e.g., self-esteem, emotion regulation, theory of mind skills, executive function skills); family level (e.g., parental attachment, parental mental	• No third factor<br>• Un-modifiable (once child at primary school) third factors – e.g., gender, age, antenatal conditions (e.g., maternal smoking), toxins<br>• Gene studies<br>• brain /FMRI studies<br>• Institutional care/adoption<br>• Fatty acids, medications

	health); community level (e.g., peer relationships, classroom modifications).<br>• Motor coordination<br>• BMI/weight/exercise	• Attentional difficulties
**Context**	• Any country<br>• Kids in mainstream or special schooling<br>• Alphabetic languages	• Non-alphabetic languages

Table S2: An example of a full search

APA PsycInfo <1806 to July Week 1 2021>

#	Query	Results from 11 Jul 2021
1	(Child* or teen* or adolescen* or youth or young).mp. [mp=title, abstract, heading word, table of contents, key concepts, original title, tests & measures, mesh]	1,224,590
2	exp Dyslexia/	6,969
3	(dyslexi* or "poor reader*" or "reading abilit*" or "reading skill*" or "reading achievement" or "reading difficult*" or "reading disorder*" or "reading impair*" or "reading delay" or "reading defici*" or "reading disabilit*" or "literacy difficult*" or "learning disorder" or "struggl* adj3 read*").mp. [mp=title, abstract, heading word, table of contents, key concepts, original title, tests & measures, mesh]	38,823
4	exp Internalizing Symptoms/ or exp Externalizing Symptoms/	1,227
5	(internali?ing or "somatic complaint*" or anxious or anxiety or depression or depressed or "emotion* difficult*" or "emotion* problem*" or psycho?social or socio?emotion* or withdrawn or "mental health" or hyperactiv* or impulsiv* or externali?ing or "conduct problem*" or "behaviour* problem*" or "behavior* problem*" or aggress*).mp. [mp=title, abstract, heading word, table of contents, key concepts, original title, tests & measures, mesh]	934,610
6	2 or 3	38,823
7	4 or 5	934,610
8	1 and 6 and 7	2,296
9	limit 8 to (peer reviewed journal and english language)	1,523

(Child* or teen* or adolescen* or youth or young).mp. [mp=title, abstract, heading word, table of contents, key concepts, original title, tests & measures, mesh]
exp Dyslexia/
(dyslexi* or "poor reader*" or "reading abilit*" or "reading skill*" or "reading achievement" or "reading difficult*" or "reading disorder*" or "reading impair*" or "reading delay" or "reading defici*" or "reading disabilit*" or "literacy difficult*" or "learning disorder" or "struggl* adj3 read*").mp. [mp=title, abstract, heading word, table of contents, key concepts, original title, tests & measures, mesh]
exp Internalizing Symptoms/ or exp Externalizing Symptoms/
(internali?ing or "somatic complaint*" or anxious or anxiety or depression or depressed or "emotion* difficult*" or "emotion* problem*" or psycho?social or socio?emotion* or withdrawn or "mental health" or hyperactiv* or impulsiv* or externali?ing or "conduct problem*" or "behaviour* problem*" or "behavior* problem*" or aggress*).mp. [mp=title, abstract, heading word, table of contents, key concepts, original title, tests & measures, mesh]
2 or 3
4 or 5
1 and 6 and 7
limit 8 to (peer reviewed journal and english language)

Table S3. Individual, Family, and Community-level factors associated with internalising/externalising symptoms among children with word-reading difficulties (dyslexia)

*** Due to the referencing requirements of this journal the reference number for the studies in this table are different to those in Table 1 in the body of the article.**

Author (Date)	Title	Aim of study	Population	Learning/Reading Difficulties	Context	Mental Health Concept	Third factor of interest	Methodology	Key findings
Abarrate (1993) Dissertation[1]	Behavior problems and social competence in attention deficit hyperactivity disorder children with and without a learning disorder	To investigate how children with ADHD with and without learning difficulties differ in relation to behaviour problems and social competence	Children aged 7-12 years (n = 26 with ADHD and n = 26 ADHD with comorbid learning difficulties)	Learning disorder based on IQ-performance discrepancy criteria using standardised measures and/or placement in Special Education class	Lower Coachella Valley, USA	Internalising/externalising symptoms measured by teacher report with the relevant subscale of the Child Behaviour CheckList (CBCL; Achenbach & Edelbrock, 1984)	**Community-level:** children's social problems (referred to as social competence) measured by teacher-report with the social problems sub-scale of the CBCL	Cross-sectional. Data was analysed with ANOVA / MANOVA. The analysis appears to have been confined to the social problems and externalising symptoms (not internalising) subscales of the CBCL	Results suggest that coexisting learning difficulty is the differentiating factor in determining whether a child with ADHD has poorer social competence and more externalising symptoms
Aitken et al., 2020[2]	Profiles of co-occurring difficulties identified through school-based screening	To investigate patterns of co-occurrence among five common childhood difficulties: inattention/hyperactivity, internalising symptoms, externalising symptoms, peer problems and reading difficulties	501 children (age range 6-9 years; 254 girls) were recruited from seven schools. 97% of the sample spoke English as their first language. Eligibility for participation was based on being in a mainstream class, and not having any sensory or physical impairments	A reading difficulties group was determined based on scores in the bottom quartile of the Dynamic Indicators of Basic Early Literacy Skills Oral Reading Fluency Task (Good & Kaminski, 2002)	Participants were recruited from one suburban and one rural school board in Ontario Canada. 25% of the children were in a French immersion class in which most of the instruction is in French.	Internalising/externalising symptoms were reported on by parents with the Strengths and Difficulties Questionnaire (SDQ; Goodman, 1997)	**Community level:** Peer problems measured by parent and teacher report with the peer problems sub-scale of the SDQ	Longitudinal. The data was collected at two time-points 1 year apart and analysed using latent class analysis	In the present study no co-occuring difficulties with reading, peer problems or internalising/externalizing symptoms were present

Author (Date)	Title	Aim of study	Population	Learning/Reading Difficulties	Context	Mental Health Concept	Third factor of interest	Methodology	Key findings
Alexander-Passe (2006)[3]	How dyslexic teenagers cope: An investigation of self-esteem, coping, and depression	To investigate how adolescents with dyslexia cope with their difficulties and how this relates to their self-esteem and depression	19 adolescents (12 male *M-academic year* = 11.17; 7 female, *M-academic year* = 11.86)	Recruitment occurred through dyslexia associations and an educational psychologist. No further description of criteria for dyslexia were provided	London, United Kingdom	Depression was measured by self-report with the Beck-Depression Inventory (BDI-II; Beck et al., 1996)	**Individual-level**: Adolescents reported on their self-esteem with the Culture-free Self-Esteem Inventory (CFSEI; Battle, 1992) and their coping strategies with the Coping Scale for Stressful Situations (adolescent versions) (Endler & Parker, 1999)	Cross-sectional. Results on variables of interest were compared to the findings of other studies	Results suggest that girls with dyslexia may be particularly susceptible to depression and low self-esteem and employ more emotional and avoidant coping strategies than boys
Al-Yagon (2009)[4]	Comorbid LD and ADHD in childhood: Socioemotional and behavioural adjustment and parents' positive and negative affect	To examine a variety of risk and protective factors for socio-emotional difficulties among children with co-morbid ADHD/LD relative to those considered to have typical development	The sample consisted of 118 father-mother-child (8-12 years) triads; 59 families with children with co-morbid ADHD/LD (42 boys, 17 girls) and 59 with a child with typical development (40 boys, 19 girls)	All children in the co-morbid ADHD/LD group had been formally diagnosed prior to participating in the study. Diagnostic criteria were reported as being consistent with DSM-4 criteria. Sub-type of learning difficulty was not described	Children were attending public schools in the urban areas of central and southern Israel	Internalising/externalising symptoms measured by father and mother report on the Child Behaviour CheckList (CBCL; Achenbach, 1991).	**Individual-level:** Children reported on their coping and resilience with the Children's Sense of Coherence Scale (Margalit & Efranti, 1995; the Effort Scale (ES; Lackaye & Margalit, 2006); the Children's Hope Scale (CHS; Lackaye & Margalit, 2006). **Family-level**: Children reported on child-parent relationships with the Attachment Security Scale (ASS; Kers et al., 1996) and parents reported on their own confidence and mental health with the Affect Scale (AS; Moos et al., 1987**). Community level:**	Cross-sectional. A group comparison (children/parents with/without disability) analysis was followed by an investigation of the contribution of the various risk and protective factors to children's socio-emotional wellbeing	Relative to children with typical development, children with ADHD/LD self-reported more loneliness, lower sense of coherence (resilience) and less hope. Parents of these children, relative to those with typical development, also reported more socio-emotional and behavioural difficulties (internalising and externalising) among their children. Father's negative affect and depression, and mother's negative affect were associated with higher levels of child internalising/externalising symptoms. Children’s attachment to parents did not appear to be associated with their internalising/externalising symptoms in this sample

Author (Date)	Title	Aim of study	Population	Learning/Reading Difficulties	Context	Mental Health Concept	Third factor of interest	Methodology	Key findings
							Children completed the Loneliness and Social Dissatisfaction Scale (LSDS; Asher et al., 1990)		
Al-Yagon (2014)[5]	Child-mother and child-father attachment security: links to internalizing adjustment among children with learning disabilities	To investigate the role of children's attachment to their father and mother in explaining internalising symptoms among children with learning disabilities relative to those with typical development	The sample consisted of 107 children with learning difficulties (52 girls and 54 boys) and 98 children (56 girls and 42 boys) with typical development aged 8-12 years and their parents	All children in the LD group had been formally diagnosed prior to participating in the study. Diagnostic criteria were reported as being consistent with DSM-4 criteria. Sub-type of learning difficulty was not described	Children were recruited from 7 elementary schools in urban areas of central Israel	Fathers and mothers reported on their child's internalising symptomology with the internalising sub-scale of the Child Behaviour CheckList (CBCL; Israeli translation)	**Individual-level**: Children reported on their coping strategies (sense of coherence, hope, and effort (e.g., item "I don't give up even when it is difficult for me") with the Children's Sense of Coherence Scale (Margalit & Efranti, 1995) and Children's Hope and Effort Scale (Snyder et al., 1997; Lackaye & Margalit, 2006**)**. **Family-level**: Children also reported on child-parent relationships with the Attachment Security Style (Kerns, Klepac & Cole, 1996). **Community-level:** loneliness and social (dis) satisfaction measured with the Loneliness and Social Dissatisfaction Scale (Asher et al., 1990)	Cross-sectional. Group differences on all variables were analysed followed by Structural Equation Modelling to analyse the influence of mother and father attachment on all the child variables (loneliness, internalising, coping)	Children with learning difficulties had more parent reported internalising symptoms, more self-reported loneliness, and poorer coping (effort, hope, and sense of coherence) than typically developing peers. The child and parent-reported internalising symptoms did not correlate in either group. However, findings suggest that children with learning difficulties may be more vulnerable to insecure attachment with their parents and indicate the possible protective function of secure relationships with mothers and fathers
Al-Yagon (2016)[6]	Perceived close relationships with parents, teachers, and	To compare adolescent's attachment relationships with	280 adolescents (15-17 years, 154 girls), 90 with a learning	Sub-samples of children with learning difficulties were all	Children came from regular public high schools in urban	Children self-reported on their mental health with the Hebrew adaptation of the Affect Scale (Moos et al., 1987) and the youth	**Family and Community-level**: Intergroup differences in 3 forms of	Cross-sectional. Data was analysed via multigroup structural	High quality of perceived friendship with a best friend seemed particularly important for the mental health (both

Author (Date)	Title	Aim of study	Population	Learning/Reading Difficulties	Context	Mental Health Concept	Third factor of interest	Methodology	Key findings
	peers: Predictors of social, emotional, and behavioural features in adolescents with LD or comorbid LD and ADHD	parents, teachers, and friends and how these are related to children's mental health	difficulties diagnosis, 91 with combined ADHD and learning difficulties, and 99 classified as typically developing	formally diagnosed with an SLD in accordance with DSM-4 criteria. Subtype of learning difficulty was not described	areas of Israel. First language wasn't mentioned but measures used were all Hebrew language	version of the Child Behaviour CheckList (CBCL; Achenbach, 1991)	attachment relationship (parents, teachers, peers) were investigated: Child-parent relationship (using the Attachment Security Style; Kerns et al., 1996), child-teacher relationship (using the Children's Appraisal of Teacher as a secure Base Scale (Al-Yagon & Mikulincer, 2006) and friendship (using the Friendship Quality Questionnaire; Parker & Asher, 1993)	equation modelling	internalising and externalising) of children with learning difficulties (with and without ADHD). Having a caring teacher was associated with higher levels of positive affect among children with learning difficulties. Children with learning difficulties, compared to controls, appeared more vulnerable to the effects of insecure attachment to their mothers in terms of their mental health than the other groups
Antshel & Guy-Ronald (2006)[7]	Maternal stress in nonverbal learning disorder: A comparison with reading disorder	To investigate mothers' stress and coping in association with children's mental health among children with two subtypes of learning difficulty: reading disorder and non-verbal learning disorder	75 mother-child dyads (8-11 years, 51 boys) including 31 children with a diagnosed reading disorder, 21 with non-verbal learning disorder and a "typically developing" control group matched by age, gender and IQ	Inclusion criteria to the reading disorder sub-group were that the children met DSM-4 criteria for a reading disorder	USA. The majority of participants were European American and all spoke English as a first language	Children's social, emotional, and behavioural wellbeing was measured by parent and teacher report using the relevant versions of the Behaviour Assessment for Children (BASC; Reynolds & Kamphaus, 1992).	**Family-level**: Mother's self-reported parenting stress, mental health, and perceived levels of social support measured respectively by the Parenting-Stress Index-Short Form (PSI-SF; Abidin, 1990) the Symptom-Checklist 90-Revised (SCL-90-R; Derogatis, 1983) and Social Support Questionnaire (SSQ; Sarason et al., 1983)	Cross-sectional. Regression analysis was used to investigate the contribution of the maternal variables to children's internalising and externalising symptoms by group (reading difficulties vs non-verbal learning disorder vs typical development)	Mothers of children with reading disorder reported higher levels of general distress than the other 2 groups and were more likely to report their child's behaviour as "difficult" relative to those who had children with no learning disabilities. Among those with reading difficulties, children's internalising symptoms were not related to their mother's level of stress but were associated with mother's mental health concerns and their perceived levels of social support
Baumeister et al., (2008)[8]	Peer victimization in children	To investigate psychosocial correlates of	77 children and adolescents with learning	All participants had a learning difficulties	USA	Internalising/externalising symptoms measured by parents with the Child Behaviour	**Community-level**: Peer victimisation was measured by	Cross-sectional. Pearson correlations were used	Peer victimisation was positively correlated with parent reports of children's internalising

Author (Date)	Title	Aim of study	Population	Learning/Reading Difficulties	Context	Mental Health Concept	Third factor of interest	Methodology	Key findings
	with learning disabilities	peer victimisation among children with learning disabilities	difficulties (5-18 years, *Mage* = 11.3 years, 68 males) referred for either learning difficulties diagnosis or psychiatric assessment	diagnosis but criteria for diagnosis were not further described. The researchers accessed their clinical case files to conduct the research		Check-List (CBCL; Achenbach, 1991) and behaviour/attentional difficulties with Connors Parents Rating Scale-Revised (Goyette et al., 1978). Children reported on depression and anxiety with the Children's Depression Inventory (CDI; Kovacs, 1992) and the Revised Children's Manifest Anxiety Scale (RCMAS; Reynolds & Richmond, 1978)	parent report with McCloskey and Stuewig's (2001) peer victimisation scale	to analyse associations between variables of interest	and externalising symptoms and children's self-reports of depression symptoms
Benassi et al., 2020 [9]	Impaired school well-being in children with Specific Learning Disorder and its relationship to psychopathological symptoms	To investigate the association between school well-being and children's mental health from the point of view of children and mothers	72 children (8-10 years, 52 without learning difficulties and 20 with learning difficulties) and their mothers. The SLD group comprised 16 males and 4 females and the control group comprised 27 males and 25 females	The learning difficulties diagnosis complied with ICD-10 guidelines. The number of children with reading difficulties was not reported	The children were recruited from 3 traditional schools within the Moderna district of Italy	Parents reported on children's internalising/externalising symptoms with the Child Behaviour CheckList (CBCL; Achenbach, 1991; Achenbach & Rescorla, 2001).	**Community-level:** school wellbeing was assessed by child and parent report with the Questionnaire on School Well-being (QBS; Tobia & Marzochi, 2015)	Cross sectional. General linear models were applied	There were closer associations between poor school wellbeing (struggles with learning and emotional problems at school) and higher levels of parent-reported internalising/externalising symptoms among children with learning difficulties, relative to those without any known learning difficulty, suggesting that school wellbeing may be a particularly salient factor in the mental health of children with learning difficulties
Blicher et al. (2017) [10]	The role of trait anxiety and preoccupation with reading disabilities of children and their mothers in predicting children's reading comprehension	To investigate how reading comprehension in children with reading disability is predicted by trait anxiety, and preoccupation with reading disability (both child and mothers)	The sample consisted of 88 children (grade 3-5, 55.7% boys) with word-reading difficulties and their mothers	Children identified by school as having difficulties with basic reading and reading comprehension. The researchers then validated this by assessing children	The children were all in regular elementary school classes in Israel and were recruited from 12 schools	Children's trait anxiety was measured by self-report with the Hebrew version of the State-Trait Anxiety Inventory (STAI; Speilberger et al., 1975)	**Individual-level**: Children's pre-occupation (self-consciousness) with their reading difficulties was measured with the Preoccupation with Reading Disability Questionnaire (PRDQ; Shaney et al., 2011). **Family-level:**	Cross-sectional. Pearson correlations followed by an analysis of direct and indirect effects using the PROCESS macro (Hayes, 2013)	Children's reading comprehension could be explained by their word reading ability, anxiety and their mother's preoccupation with their child's reading difficulties, but not maternal anxiety. Of interest to this review, children's trait anxiety was positively associated to their own and their mothers' preoccupation with their reading difficulties

Author (Date)	Title	Aim of study	Population	Learning/Reading Difficulties	Context	Mental Health Concept	Third factor of interest	Methodology	Key findings
				on word-reading fluency			Mother's anxiety was measured with the STAI and their pre-occupation with their child's reading difficulties with a parent version of the PRDQ		
Boetsch et al., 1996 [11]	Psychosocial correlates of dyslexia across the life span	Studies 2, 3 and 4 are of interest to this review as they focused on children and adolescents. The aim was to examine the psychosocial functioning of children and adolescents with dyslexia	The researchers chose three different samples of children (aged 10-12 years): a clinic-referred sample, a community sample, and a twin sample. All studies incorporated a "typically developing" control group	A formal dyslexia diagnosis with a discrepancy between reading/spelling performance and what would be expected based on their age, IQ, and educational experience	USA	Children reported on their depression symptoms with the Dimensions of Depression Profile for Children and Adolescents (Harter & Nowakowski, 1987); and the Children's Depression Inventory (CDI; Kovacs, 1981). ADHD symptomology was measured in all three studies by the ADHD subscale of the Diagnostic Interview for Children and Adolescents (DICA; Herjanic, 1982).	**Individual-level**: Children reported on their self-worth with the Self-Perception Profile for Learning Disabled Students (Rennick & Harter, 1988). **Family/Community-level:** Children's perception of social support from friends, parents, teachers, and classmates (study 2 only) was measured with the Social Support Scale for Children (Harter, 1985).	Cross-sectional. For studies 2 and 4 two-way MANOVAs were conducted (dyslexic and non-dyslexic groups) and age (chid and adolescent). For study 3 between-groups analyses consisted of paired sampleT-tests to compare each pair of twins	Across all samples, children, and adolescents with dyslexia, relative to controls, reported lower global self-worth, lower academic self-competency, more depression symptomology and more (parent and child reported) ADHD symptoms. In clinic referred samples at least, children and adolescents with dyslexia reported being significantly less supported by parents, teachers, and peers than their typically achieving peers. Parents appeared to be more aware of externalising than internalising symptoms. There were no significant differences related to age or gender in the mental health variables
Bonifacci et al. (2016)[12]	Specific Learning Disorders: A look inside children's and parents' psychological well-being and relationships	To investigate the relationship between parent and child psychological profiles among a sample of children with learning difficulties	34 triads (child, father, and mother) participated. A learning difficulties sub-sample of 17 children (Mage = 10.5 years +/- 1.6 yrs, 13 males) and a control group of 17 without	All children in the learning difficulties sub-sample had received a formal diagnosis of SLD-mixed type (i.e., at last two difficulty domains across reading, writing, and	Italy. Families of children with learning difficulties were recruited through a specialist assessment and intervention clinic	Both children and parents reported on children's internalising symptoms using the Test of Anxiety and Depression (TAD; Newcomer et al., 1995)	**Individual-level**: Children's self-esteem was measured by the Self-esteem Multidimensional Test (TMA; Bracken, 2003). **Family-level:** Parental stress was measured by the Parenting Stress Index (PSI; Abidin, 1995), parental	Cross-sectional. MANOVA's with group (SLD and control) and parent (mother and father) as between-subject factors	Parents of children with learning difficulties, relative to those without, reported higher levels of parenting stress but did not differ regarding mental health concerns. Children with learning difficulties reported significantly lower scholastic and inter-personal self-esteem but not higher levels of anxiety or depression, although they were rated as more anxious and

Author (Date)	Title	Aim of study	Population	Learning/Reading Difficulties	Context	Mental Health Concept	Third factor of interest	Methodology	Key findings
			learning difficulties (Mage = 10.7 years, 9 males) and their parents participated	mathematics). The number with reading difficulties was not described			mental health measured by the Symptom-Checklist 90-Revised (SCL-90-R; Derogatis, 1983) and parenting practices (e.g., discipline styles) measured by the Parenting Scale (PS; Arnold et al., 1993)		depressed by their parents. Across the groups, there were associations between higher levels of children's depression and greater maternal stress, psychological difficulties, and ineffective disciplinary styles
Bonifacci et al., (2020)[13]	Rumination and emotional profile in children with Specific Learning Disorders (SLD) and their parents	To study whether children with SLD and their parents ruminate more than typically developing peers and whether rumination is associated with child and parent mental health concerns	50 children and their parents (50 mothers and 50 fathers) were recruited, half with confirmed SLD (*Mage* = 10.08 years +/- 1.15 yrs) and a control group without SLD (*Mage* = 9.88 years +/- 0.53 yrs) matched by age and gender	Diagnosis was by full neuropsychological assessment and conformed to ICD-10 criteria. 24% of the SLD group had a diagnosis of dyslexia, 4% dysorthorgraphia (spelling), 16% dyscalculia and 56% had mixed learning disorders	Italy	Children reported on their anxiety with the Multidimensional Anxiety Scale for Children (MASC; March et al., 1997). Parents reported on their children's psycho-social functioning with the Strengths and Difficulties Questionnaire (SDQ; Goodman, 1997)	**Individual-level**: Children reported on their coping strategies with the Response Style Questionnaire (CRSQ; Abela et al., 2004) and 3 emotional competencies: emotional control, emotional self-awareness, and situational responsiveness with the Emotion Regulation Index for Children and Adolescents (ERICA; MacDermott et al., 2010) and rumination with the Kid's Rumination Interview (KRI; Baiocco, R. et al., 2017) and their prosocial behaviour was assessed by parents with the relevant sub-scale of the SDQ. **Family-level**: parents reported on	Cross-sectional. MANOVAs were used to investigate group differences (SLD vs control) on the parent-reported child variables. Independent group Ttests (SLD vs control) were used to analyse the parent data. Pearson correlation analysed parent variables in association with child rumination but not child mental health	After Bonferroni corrections were applied there were no significant group differences in internalising/externalising symptoms, prosocial behaviour, peer problems, coping strategies, emotional competencies or generalised ruminative tendencies. However, children with SLD reported more rumination on a vignette related to teasing by peers and their mothers reported higher depression and parent's reported higher ruminative tendencies

Author (Date)	Title	Aim of study	Population	Learning/Reading Difficulties	Context	Mental Health Concept	Third factor of interest	Methodology	Key findings
							their emotion regulation with the Italian adaptation of the Rumination Response Scale (RRS; Nolen-Hoeksema& Morrow, 1989) and their mental health with a battery of standardised measures including the Penn State Worry Questionnaire (PSWQ; Meyer et al., 1995) **Community-level:** children's peer problems were measured by parent report with the relevant sub-scale of the SDQ		
Boyes et al., (2020)[14]	Correlates of externalising and internalising problems in children with dyslexia: An analysis of data from clinical casefiles	To examine a range of hypothesised factors associated with internalising and externalising symptoms among children with dyslexia	The case files of 450 children and adolescents with dyslexia (age range 6-17 years, 58% male) were analysed	All formally diagnosed with dyslexia according to DSM-5 (APA, 2013) criteria	Australia. The case files were provided by the Dyslexia SPELD Foundation (DSF) a leading Western Australian provider of services to children and families with learning difficulties	Was assessed twofold. Firstly, by parent report on a single open-ended question during diagnostic interview. This was then dichotomised (0= no behaviour difficulties and 1 = behaviour difficulties) for analysis. Secondly, further open-ended sections of the parent questionnaire were searched for reference to other emotional difficulties such as mood problems	**Individual-level**: Self-esteem, emotion regulation, social skills**. Family-level:** parent-child relationship**. Community-level**: peer problems, bullying victimisation, school support, child-teacher relationship, all measured by survey items in clinical interviews with parents	Cross-sectional data was analysed using two multiple regressions: one for internalising and one for externalising difficulties. Mediation models were then tested	Among children with dyslexia, inverse associations between self-esteem, emotion regulation, and social skills and positive associations between bullying victimisation and internalising and externalising symptoms were found. Additionally, peer relationship difficulties were indirectly associated with greater internalising /externalising symptoms via low self-esteem and emotion regulation difficulties. School and family factors were not significant in the multivariate analysis but teacher/parent-child relationship difficulties did correlate

Author (Date)	Title	Aim of study	Population	Learning/Reading Difficulties	Context	Mental Health Concept	Third factor of interest	Methodology	Key findings
									with mental health concerns in the bi-variate analysis
Camilleri et al. (2019)[15]	"They labelled me ignorant": Narratives of Maltese youth with dyslexia on national examinations	To explore the experiences of students with dyslexia within the context of sitting national examinations	Research participants were 8 Maltese adolescents (aged approx. 15-16 years)	All had been formally assessed as having dyslexia	Malta	Stress, anxiety, in particular "test anxiety" was explored	**Community-level**: Misunderstandings and lack of knowledge about dyslexia leading to inappropriate learning/assessment accommodation	Qualitative design. Participants were interviewed and transcripts were analysed thematically using a discourse analysis approach	The researcher identified that a perception of a lack of understanding about the needs of people with dyslexia contributed to stress and anxiety in this high-stakes assessment situation
Capozzi et al. (2008)[16]	Psychiatric comorbidity in learning disorder: Analysis of family variables	To evaluate the role of parenting style on the mental health of children with Learning Disability (LD)	The sample consisted of 56 children (7-12 years, 44 boys and 12 girls) and their parents	Children were all diagnosed with a learning disability. The number with dyslexia was not described	Italy. Children were recruited via a health clinic where they had all been assessed for school-related problems	Parents reported on children's internalising/externalising symptoms with the Child Behaviour CheckList (CBCL; Achenbach, 1991)	**Family-level**: Parent's reported on the child-parent relationships with the Attachment Style Questionnaire (ASQ; Feeney et al., 1994) and parenting practices with the Family Attitudes Questionnaire - Italian Version (FAQ; Duszynski, 1974; Pecci et al., 2003)	Cross-sectional design. Stepwise regressions were used to analyse the data with internalising and externalising symptoms as outcome variables and the various family factors as predictor variables	"Pathological" levels of internalising, relative to externalizing, symptoms were reported by parents. Children's attachment insecurity with parents (differentially for father and mothers) was associated with higher levels of internalising/externalising symptoms
Carroll et al. (2005)[17]	Literacy difficulties and psychiatric disorders: Evidence for comorbidity	To investigate the nature and specificity of links between specific literacy difficulties and psychiatric disorder	A representative sample of 5752, 5–15-year-old British (England, Scotland and Wales) children and adolescents (68 girls and 221 boys classified with learning difficulty)	The 5% of children (9 years and older) whose reading, or spelling scores were furthest below their predicted scores based on their vocabulary scores	United Kingdom. The researchers gathered their data from the 1999 national survey of child mental health carried out by the UK office for National Statistics in 1999	Parents and teachers reported on internalising/externalising symptoms with the Strengths and Difficulties Questionnaire (SDQ; Gooman, 1997) and the childhood psychiatric disorders with the Development and Well-being Assessment DAWBA; Goodman et al., 2000). In addition, children aged 11 years and older completed the Short Mood and Feelings Questionnaire (SMFQ; Angold et al., 1995) which contains items reflecting the symptoms of depression	**Family-level**: Of interest to this review parents reported on family functioning with the McMaster Family Activity Device (Miller et al., 1985) and mothers reported on their level of maternal distress using the General Health Questionnaire (GHQ-12; Godlberg & Williams, 1988)	Cross-sectional design. Data was analysed with ANOVA and regression models	Attentional difficulties appeared to mediate links between literacy difficulties and higher levels of externalising symptoms as well as children's own reports of "low mood", but anxiety could not be explained by co-occurring attentional difficulties. Of note to this review, in this study literacy difficulties were not associated with higher levels of maternal distress or poorer family functioning

Author (Date)	Title	Aim of study	Population	Learning/Reading Difficulties	Context	Mental Health Concept	Third factor of interest	Methodology	Key findings
Casey et al., 1992[18]	Impaired emotional health in children with mild reading disability	To investigate the impact of reading difficulties on children's self-esteem and emotional health	28 children (aged 8-12 years) met criteria for the reading disorder group. A control group of 39 without any known learning difficulty were recruited through routine health screening	All children with reading difficulties met DSM-3 criteria for developmental reading disorder	USA. The students with reading difficulties were all attending a tailored school summer camp, and were described as experiencing "mild" reading difficulties and coming from supportive home environments of high SES status	Parents reported on their children's mental health with the Rand Mental Health Index (Eisen et al., 1980) which has three sub-scales: anxiety, depression, and positive wellbeing. Parents also reported on children's internalising/externalising symptoms with the Child Behaviour CheckList (CBCL; Achenbach, 1978/79 versions)	**Individual-level:** Both parents and children reported on children's self-perceptions using the relevant versions of Harter's Self-Perception Profile (Harter, 1982)	Cross sectional. Group comparison study	Children with dyslexia, relative to peers without learning difficulties, reported lower levels of scholastic self-competence but not other domains of self-competence. No significant group differences in internalising/externalising behaviour on the CBCL were found, however, higher levels of anxiety and lower levels of well-being were reported by parents of children with dyslexia, relative to those without learning difficulties, on the Rand Mental Health Survey. The researchers concluded that children with reading difficulties were at risk of poor self-esteem and poor emotional health despite having supportive home environments
Cen & Aytac (2017)[19]	Ecocultural perspective in learning disability: Family support resources, values, child problem behaviors	To investigate associations between mother's perceived social support resources, values, and children's "problem behaviors" (internalising/externalising symptoms)	Ninety children and adolescents with learning difficulties (7-14 years, 42 girls and 48 boys), their mothers and teachers	All diagnosed with learning difficulty through previous psych-educational assessments. No break-down of numbers with reading difficulty reported	Turkey	Internalising/externalising symptoms measured by teacher-report with the Child Behaviour Problem Scale (Kanner, 2007)	**Family-level**: Mother's reported on their level of family social support with the Family Support Scale (Kanner, 2003) and their parenting values with the Portrait Values Questionnaire (PVQ; Schwartz et al., 2001)	Cross sectional. Pearson correlation, regression analysis, and Haye's moderation analysis were conducted	After controlling for severity level of learning difficulty (teacher reported) there was some evidence of parental values (openness to change and "conservation" (secure home environment) and aspects of mothers' perceived social support (informational support) being associated with less internalising symptoms among children and mother's perceived emotional support being associated with less externalising symptoms among children
Chiapeddi & Baschenis (2016) [20]	Specific learning disorders and anxiety: A matter of	To investigate associations between anxiety among children	20 children and adolescents (aged 8-13) with SLD and 32	SLD diagnosed according to ICD-10 criteria. No description	Italy. The SLD group were recruited via a health centre.	Children reported on their anxiety with the Anxiety sub-scale of the Self-administered Psychiatric Scales (SAFA-A; Cianceitti et	**Community-level**: Perceived support from teachers was measured by a	Cross-sectional data was analysed by Mann Whitney Utest	As predicted children with SLD reported significantly higher anxiety than the control group. Further, children with SLD who

Author (Date)	Title	Aim of study	Population	Learning/Reading Difficulties	Context	Mental Health Concept	Third factor of interest	Methodology	Key findings
	school experience?	with learning difficulties and their school experience (namely perceived level of support from their teachers)	same-aged typically developing peers	of how many children had dyslexia	The control group were recruited from one local school	al., 2001) which measures: generalised anxiety, worry about the future, social anxiety, separation anxiety, and school-related anxiety	single survey item. Children with SLD were asked: "Do you think that your teachers were able to understand your disorder and help you?"	and Spearman's Rho	perceived their teacher as understanding and caring had lower levels on all domains of anxiety
Claessen et al., (2020)[21]	Educators' perceptions of the impact of reading difficulties for young people	To further understanding about risk and protective factors for the wellbeing of children with reading difficulties	20 educators who all had a minimum of 2 years classroom experience and currently working with children aged 5-18 with reading difficulties	The children described had to have been identified as having persistent reading difficulties	Perth, Western Australia	Mental health was constructed as comprising of both internalising and externalising symptoms and explored thematically	**Individual-level**: responses to diagnosis, in particular feelings of shame. **Community-level**: social stigma (i.e., feeling "different")	Qualitative. A phenomological approach was taken and Braun and Clarke's (2006) approach to thematic analysis used to analyse the data	Stigma related to feelings of shame and difference were identified as possible risk factors to children's mental health
Dahle et al. (2011)[22]	Coexisting problem behaviour in severe dyslexia	To better understand internalising and externalising symptoms in dyslexia in different settings (e.g., home/school)	A group of 70 children and adolescents (59 boys, 11 girls, Mage = 150.49 months) with severe dyslexia and a control of 70 children (pair-wise matched on age, gender, cognitive level, and rural/urban context) without reading difficulties (Mage = 101.31 months)	Diagnosis of dyslexia based on phonological difficulties. Nine children in the reading difficulties group also had an ADHD diagnosis and 1 had a diagnosis of Tourette's syndrome	Norway.	Parent, teacher, and child reports using the Child Behaviour CheckList (Achenbach & Rescorla, 2001)	**Community-level:** Of interest to this review, children's social problems were also assessed from multiple perspectives with the CBCL	Cross-sectional. Group comparison design.	Dyslexia was strongly associated with higher levels of internalising symptoms in this sample, more than externalising symptoms. Parents reported higher levels of internalizing symptoms than teachers. Teachers, but not the children themselves, appeared to identify social problems among the students with dyslexia and all were boys. The study highlighted the importance of considering the context (school/home) when considering children's socio-emotional difficulties
Dahle & Knivsberg, (2014)[23]	Internalizing externalizing, and attention	To investigate dyslexia-related group differences in	Twenty-six 9–10-year-old children with dyslexia (Mage = 9	Dyslexia was determined based on poor performance	Students all came from mainstream	Internalising/externalising behaviour reported by teachers and parents with the Child	**Community-level:** Social problems also assessed using teacher and parent	Cross-sectional	Parents and teachers reported a higher level of internalising and externalising difficulties in the dyslexia group relative to

Author (Date)	Title	Aim of study	Population	Learning/Reading Difficulties	Context	Mental Health Concept	Third factor of interest	Methodology	Key findings
	problems in dyslexia	behavioural/emotional problems	years and 8 months) and 26 without (Mage = 9 years and 10 months)	(lowest 20% of students) on standardised tests of word reading and spelling	schools in rural Norway	Behaviour CheckList (CBCL; Achenbach & Rescorla, 2001)	reports on the social problems sub-scale of the CBCL		controls but only the teacher ratings reached significance. Teachers rated the children with dyslexia as having more social problems and aggressive behaviors than children without any known learning difficulty
D'Amico & Guastaferro, (2017) [24]	Emotional and meta-emotional intelligence as predictors of adjustment problems in students with Specific Learning Disorders	To investigate the effect of severity level of SLD and emotional intelligence on the psycho-social wellbeing of adolescents with a diagnosed SLD	34 Italian adolescents (14-19 years, 25 male, Mage=16 years) with an SLD and their parents participated	All adolescents met DSM-4 criteria (APA, 1994) for an SLD. Of these, 20 of the total sample self-reported difficulties in reading	Italy. Participants were contacted through their schools and assessed using the Italian language versions of all measures	Internalising, externalising and total "problem behaviour" was measured by children with the Italian version of the Youth Self-Report (aged 11-18 years) and parent version of the Child Behaviour CheckList (CBCL; Achenbach, 1991)	**Individual-level:** Emotional intelligence was measured by the Intelligenza Emotiva: Ability, Credenze e Concetto do Se Mata-Emotiva (IE-ACCME, D'Amioca, 2013) a self-report scale with 3 sub-scales purporting to measure: beliefs about emotions; self-concept about emotional abilities (e.g., "I am able to identify the emotions that derive from particular physical sensations"); and emotional abilities (through responses to emotion problem vignettes) (p. 21).	Cross-sectional. Correlational analysis followed by multiple linear regressions were used to analyse the data	The results suggest that emotional beliefs, emotional self-concepts, and emotional intelligence are positively associated with the mental health (especially fewer internalising symptoms) of children with learning difficulties
DeBrew (2017)[25]	Who will help my son? A family's journey with dyslexia	A case study approach was used to explore the role of mental health nurses in the care of children with	Case study of a parent perspective. A personal account from one mother who is also a mental health nurse	The child presented with early reading difficulties and was later diagnosed with dyslexia	USA	School anxiety was explored	**Individual-level**: self-worth and **Family/community-level**: parental/teacher support. **Community-level**: Stigma and teacher (mis)understanding	Qualitative design: a single case study	A call to action for mental health nurses to be aware of the socio-emotional needs of children with dyslexia and their families. Greater school anxiety was related to teacher misunderstanding and lower self-esteem (i.e., "feeling stupid");

Author (Date)	Title	Aim of study	Population	Learning/Reading Difficulties	Context	Mental Health Concept	Third factor of interest	Methodology	Key findings
		dyslexia and their families							family support is highlighted as protective
de Lima et al. (2020)[26]	Behavior problems and depressive symptoms in developmental dyslexia: Risk assessment in Brazilian students	To compare students with and without dyslexia on behavior problems and depressive symptoms	Sixty-one children and adolescents (aged 7-14 years) including 31 with a dyslexia diagnosis and 30 without learning difficulties	The dyslexia group met DSM-5 diagnostic criteria	Brazil	Internalising/externalising symptoms measured by parent report with the Child Behaviour CheckList (CBCL; Achenbach & Rescorla, 2004) and child depression measured by self-report with the Children's Depression Inventory (CDI; Kovacs, 1981)	**Community-level:** parents also reported on children's social problems with the CBCL	Cross-sectional	Students with dyslexia, relative to those without learning difficulties, had more self-reported depressive symptoms and higher levels of parent-reported social problems and internalising and externalising symptoms
Eissa (2010)[27]	Behavioral and emotional problems associated with dyslexia in adolescence	To examine the emotional and behavioural difficulties associated with dyslexia	The sample consisted of fifty-six adolescents, 12-18 olds, 35 with reading difficulties and 21 typical readers matched by age	All adolescents in the reading difficulties group had a reading disability (dyslexia) in accordance with ICD-10 and DSM-4 criteria. The researchers noted that they all were without pre-existing psychopathology or a history of premature birth, head injury or seizure	Country of origin unknown. Participants in the reading difficulties group had typically been referred for assessment by their parent/teacher due to behavioural and/or learning concerns	Adolescents reported on their mental health with three standardised measures: The Youth Self Report of the Child Behaviour Check-List (version unstated) and the Hamilton rating scales of depression (Hamilton, 1960) and anxiety (Hamilton, 1959)	The adolescents also took part in a semi-structured interview in which they reported on; **Individual-level**: self-esteem, and **Community-level:** success/failure at school, feelings of well-being at school, the impact of dyslexia on school achievements, and peer relationships/bullying	Mixed methods design using cross-sectional qualitative and quantitative data	Adolescents with reading difficulties, relative to their typically reading peers, evidenced higher levels of both internalising (anxiety and depression) and externalising difficulties and poor overall self-esteem. Internalising and externalising symptoms correlated suggesting to the researcher that some children "act out" when anxious/depressed. The interview data suggested that poor school achievement contributed to negative self-esteem but that school was "tolerable" to most participants due to having good friends. It seemed that good friendships compensated for their academic difficulties. Some reported having been bullied due to their reading/writing difficulties
Fussell et al. (2005)[28]	Social skills and behavior problems in children with disabilities with and without siblings	To examine social skills and behaviour problems and the influence of siblings among children with a	A clinical sample of 85 children and adolescents (6-15.6 years, 58 males) and a corresponding	Of the total sample, 42 (approx. 50%) had a learning difficulties diagnosis (with or without co-	South Carolina, USA	Parents and teachers reported on children's internalising and externalising symptoms with the relevant versions of the Child Behaviour Check-List (CBCL; Achenbach, 1991)	**Individual-level**: Social skills measured by parent-report with the Social Skills Rating Scale (SSRS; Gresham & Elliot, 1990).	Cross-sectional. ANOVA was used to compare the disability groups on the key variables	Children with learning difficulties only were higher in internalising than externalising symptoms. There was no significant effect of siblings on the psycho-social measures. The influence of social skills on

Author (Date)	Title	Aim of study	Population	Learning/Reading Difficulties	Context	Mental Health Concept	Third factor of interest	Methodology	Key findings
		range of disabilities	parent were recruited from a hospital-based paediatric clinic	morbid ADHD). The number with dyslexia was not reported					internalising/externalising behaviour was not assessed
Gadeyne et al. (2004)[29]	Psychosocial functioning of young children with learning problems	To investigate psycho-social functioning among children with diagnosed learning difficulties relative to those with poor academic performance but no diagnosed learning difficulty	The sample consisted of 276 children (aged 6-7 years, 139 boys and 137 girls). The researchers formed three groups for comparison: a group with low academic achievement but no known learning disability, a group with a learning disability based on IQ-achievement discrepancy and a group with learning disability based on achievement discrepancy	The researchers formed the groups of children with Learning Disability using a well-used multi-stage procedure (Reynolds, 1994). Compared children with reading/spelling difficulties and mathematics difficulties	Children were from mainstream schools in a rural area of Belgium	Internalising/externalising symptoms measured by parent and teacher report with the Child Behaviour CheckList (CBCL; Achenbach, 1991)	As the children were quite young the researchers used pictorial measures which were based on standardised measures of all variables of interest. **Individual-level:** children's self-concept and motivation for academic work and **Community-level:** Children's views on their social relationships were measured. Teachers and parents also reported on children's social problems with the relevant subscale of the CBCL	Cross-sectional. ANOVA and MANOVA used to analyse the data	Children with reading/spelling difficulties and children with low academic achievement differed most from controls on the range of psycho-social difficulties. However, low cognitive self-concept seemed to be most related to academic achievement rather than learning disability whereas social difficulties were related to learning difficulties and children with low academic achievement seemed prone to problems with academic motivation
Gallegos et al. (2012)[30]	Anxiety, depression, and coping skills among Mexican school children: A comparison of students with and without learning disabilities	To compare the severity and risk status for anxiety and depression and coping skills among children with and without learning difficulties	260 children (130 with learning difficulties and 130 without learning difficulties, 9-12 years)	Children with learning difficulties were identified by school records which were then evaluated by a multidisciplinary team to determine	Mexico. Participants were recruited from 8 randomly selected schools	Children reported on their mental health with the Spanish versions of the Spence Anxiety Scale (SCAS; Spence, 1997) and Children's Depression Inventory (CDI; Kovacs, 1981)	**Individual-level**: Children reported on their coping strategies with the Coping Skills Questionnaire (CA; Hernandez-Guzman, 2003) a 12-item scale to measure children's interpretation	Cross-sectional. Independent samples Ttests were performed to compare children's coping skills with their levels of anxiety and depression	Children with learning difficulties reported significantly higher levels of anxiety and depression symptoms but no statistically significant group differences were reported on coping strategies

Author (Date)	Title	Aim of study	Population	Learning/Reading Difficulties	Context	Mental Health Concept	Third factor of interest	Methodology	Key findings
				group membership. The number with reading difficulties was not described			and reaction to problem situations in terms of: active coping, emotional coping, and passive/avoidant coping	symptoms by group	
Giovagnoli et al., (2020)[31]	Internalizing symptoms in developmental dyslexia: A comparison between primary and secondary school	The article reported on two studies. The second is of interest to this review. The aim of this study was to explore a range of factors associated with internalising symptoms among secondary school students with dyslexia	Study 2 included 44 adolescents (42 males) with a diagnosis of dyslexia and 54 age-matched controls (39 males) *Mage* 14.9 years	Formal diagnosis of dyslexia was required for group membership. This was then validated through reading tests administered by the researchers	Italy. All students with dyslexia came from one upper-secondary technical college	Internalising symptoms were measured by the Italian version of the Youth Self-Report (aged 11-18 years) of the Child Behaviour CheckList (YSR-CBCL; Achenbach et al., 2001)	**Individual-level**: Children reported on their coping strategies with the Italian version of the Melbourne Decision Making Questionnaire (MDMQ; Nota & Soresi, 2000) which measures 4 decision-making styles: Avoidance of problem; Procrastination in dealing with problems; Vigilance; and Hypervigilance (having "excessive alertness" toward problem situations at school (p. 461); their school and social competence with the YSR-CBCL; and their Quality of Life (QuoL) with the Clipper test (Soresi & Nota, 2003): sense of autonomy, sense of self-sufficiency, perceived self-efficacy (related to school experiences mainly).	Cross-sectional design. Several generalised linear models were used to analyse the data	Higher levels of internalising symptoms among adolescents with dyslexia were associated with peer difficulties, low levels of self-efficacy, and more hypervigilance at school. Family factors, and school wellbeing ("satisfaction with school") did not appear to be associated with internalising symptoms

Author (Date)	Title	Aim of study	Population	Learning/Reading Difficulties	Context	Mental Health Concept	Third factor of interest	Methodology	Key findings
							**Family/Community-level:** satisfaction with school experience, satisfaction in relationships and support from peers and family		
Goldston et al. (2007)[32] .	Reading problems, psychiatric disorders, and functional impairment from mid-to late adolescence	To investigate whether functional impairment and psychiatric morbidity differ between adolescents with and without reading difficulties	The sample consisted of 188 adolescents (55% male, ethnically diverse) 94 with poor reading skills and 94 with typical reading skills recruited from a larger sample of 15-year old public school students	The reading difficulties group were those with raw scores of 45 or below on the word-reading sub-test of the Woodcock-Johnson Psycho-educational Battery Revised (Woodcock and Johnson, 1990)	USA. The students were recruited from 4 urban and 2 rural schools	Psychiatric disorders (affective disorders, ADHD, conduct and oppositional disorders, substance use disorders) were assessed by child and parent report with the Schedule for Affective Disorders and Schizophrenia for School-age children Epidemiological version (K-SADS-E; Orvaschel & Puig-Antich, 1994)	**Individual-level**: Children's "functional impairment" was assessed with the Child and Adolescent Functional Assessment Scale (CAFAS; Hodges, 1997). This tool measures impairment in five domains: role functioning, behaviour towards others, mood/self-harm, substance abuse and thinking	Longitudinal design, adolescents only.	Adolescents with reading difficulties, relative to typical readers, evidenced higher rates of functional impairment and anxiety disorders even after controlling for co-morbid ADHD, in particular, generalised anxiety and social anxiety (5 times more prevalent) and difficulties in regard to "behaviour towards others" although rates of externalising disorders (conduct and oppositional disorders) did not vary significantly between the groups
Granot (2016) [33]	Socioemotional and behavioural adaptation of students with disabilities: The significance of teacher-student attachment-like relationships	To explore the extent to which children's attachment to their mothers and teachers explains the socio-emotional well-being of children with ADHD, Learning difficulties, or combined ADHD-learning difficulties	65 teacher/child dyads including 25 children and adolescents (8-14 years) with learning difficulties (LD), 20 with Attention Deficit Hyperactivity Disorder (ADHD), and 20 with both ADHD/LD	LD was diagnosed based achievement at least 2 years below that expected for grade level with no intellectual, sensory, neurological, or socio-cultural explanation. The number with reading difficulty within the LD	Northern Israel	Children's internalising/externalising symptoms were assessed by teacher report using the Teacher-Child Rating Scale (TCRS; Hightower et al., 1986)	**Family/Community-level**: Children's perception of their attachment to mothers was assessed by the ASS (Kernes et al., 1996) and attachment to teachers with the CATSBS (Al-Yagon & Mikulincer, 2006). The full name of these measures was not provided	Cross-sectional. A series of regressions were used to identify the contribution of the child-mother, child-teacher attachment relationship to children's psycho-social wellbeing	Greater maternal attachment was associated with reduced child internalising symptoms across the groups. Similarly, secure child-teacher attachment was associated with reduced externalising symptoms

Author (Date)	Title	Aim of study	Population	Learning/Reading Difficulties	Context	Mental Health Concept	Third factor of interest	Methodology	Key findings
				group was not specified					
Haager & Vaughn, 1995[34]	Parent, teacher, peer, and self-reports of the social competence of students with learning disabilities	The social competence (social skills, behaviour problems, peer relations, self-perceptions) of children with learning difficulties was compared to that of children without learning difficulties but those who were achieving at low and high academic levels	Three groups of elementary school aged children (Learning difficulties group; low achieving group; and high achieving group)	Learning difficulties had been diagnosed in accordance with DSM-3 criteria (APA, 1987). The number with reading difficulties was not described	USA. Participants were recruited from three elementary schools in a large metropolitan school district in south-eastern United States. Children in the LD group spent 50% of their time in mainstream school settings	Teachers and parents reported on internalising and externalising symptoms among children with the behaviour problems sub-scale of the relevant versions of the Social Skills Rating Scale (teacher and parent versions) (Gresham & Elliot, 1990)	**Individual-level**: Teachers, parents and children rated children's social skills with the relevant versions of the SSRS. **Community-level**: The researchers also gauged children's social experiences by asking children to nominate three classmates they liked the most and three they liked the least - this gave an indication of peer liking/popularity of each child	Cross-sectional. MANOVAs were used to analyse the data	Overall, students who were high achieving academically had fewer social and behavioural difficulties. Students with learning disabilities were rated as having more internalising symptoms (parent report), fewer social skills and more behavioural problems (teacher report) and were less liked by peers than high achieving students. However, students with learning difficulties did not appear to experience the same level of peer rejection as low achieving students without learning disability
Heiervang et al. (2001)[35]	Behaviour problems in children with dyslexia	To investigate the association between dyslexia and behaviour problems (internalising/externalising) among children with and without dyslexia	A population sample of 10–12-year-old (4th grade) children, 25 with dyslexia and 25 without any known learning difficulty	The dyslexia group was determined by the researchers based on performance on tests of spelling, single word reading, and phonological decoding strategies and exclusion criteria consistent with DSM criteria	Bergen, Norway. Children were recruited through primary schools	Parents, teachers and children completed the relevant versions of the Child Behaviour Check-List (CBCL; Achenbach, 1991) in order to assess both internalising and externalising symptoms	**Individual-level**: The factor of interest to this review is the children's pre-school spoken language ability. This was assessed by parent reported language difficulties requiring speech and language therapy and not by a diagnosis or the use of standardised tests	Cross-sectional. Data analysed with Kolmogorov-Sminov 2-sample test	Children with dyslexia had significantly more internalising symptoms according to parents and teachers; significantly more externalising problems according to parents; but no significant group differences were reported according to children themselves. Parents reported more pre-school language problems among the children with dyslexia
Heying (1987) [36] Doctoral thesis	Social competence in reading disabled boys with and	To compare boys with ADHD with and without co-	The sample consisted of three groups of children (aged 7-12	The groups were defined through the research process	The ADHD/LD group were recruited through specialist	Children's behavioural problems (internalising/externalising) were measured by mother's report with the relevant sub-scales	**Community-level**: Children's social competence was measured by the	Cross-sectional. ANOVA used to analyse data	Boys with co-morbid ADHD/RD experienced significantly more social and behavioural difficulties than boys

Author (Date)	Title	Aim of study	Population	Learning/Reading Difficulties	Context	Mental Health Concept	Third factor of interest	Methodology	Key findings
	without attention deficit disorder	morbid learning difficulties on measures of social competence and behavioural difficulties	years, all male); boys with reading disability without ADHD (n = 16); those with co-morbid RD/ADHD (n= 21); and those with no known learning or attentional difficulty (control group, n = 20)	based on standardised measures of reading and attentional difficulties	support services and the control group from one catholic school. San Diego, USA	of the Child Behaviour Check-List (CBCL) and The Conners Parent Rating Scale - Revised (CPRS-R; Goyette, et al. 1978)	social competence scale of the CBCL. This generates three scores: Activities (e.g., participation in hobbies and jobs), Social (peer relationships), and School (e.g,. academic performance)		with reading difficulties without co-morbid ADHD or boys with neither attentional or reading difficulties. No major group differences in social competence and behaviour were reported between the latter two groups. According to mothers', boys with ADHD/LD had less contact with friends and out of school organisations and poorer school performance. The findings, according to the researcher, suggest that that attentional, rather than reading difficulties, account for social and behavioural difficulties among some children
Hirsh (2014) [37] Doctoral thesis	Emotional responses to the reading difficulties experienced by young children enrolled in Title I: A qualitative study of students', teachers', and families' perspectives	To explore the reading-related dispositions, emotions, and experiences of young struggling readers from parent, teacher, and child perspectives	The sample consisted of 23 second and third grade children (and their parents and teachers) classified as poor readers by their school and receiving reading remediation	The children had been classified as struggling readers by their school and were in a pull-out program for reading remediation	USA. The research was conducted in a public elementary school in a suburb of Pittsburgh, Pennsylvania	In exploring emotional responses to learning/reading the researcher investigated children's anxiety	**Individual-level:** Children's shame and embarrassment **Community-level**: the influence of peer relationships on children's wellbeing	Qualitative design informed by grounded theory	Findings suggest that children who struggle to read often experience emotional distress, anxiety, and try to avoid reading-related tasks. Further, that a reason for the anxiety and avoidance could be fear of teasing and/or embarrassment/concerns about what their peers think of them. The researcher suggested that teachers may misinterpret children's avoidance of reading-related tasks as a negative attitude/low motivation towards reading rather than considering these social considerations
Horbach et al. (2020)[38]	Development of behavior problems in children with and without Specific	To investigate the role of ADHD as a mechanism linking childhood SLD with	The sample consisted of 292 children (at time-point 1; kindergarten, 166 boys, and	Children were classified with SLD in grade 5 if they performed below the 16th	Germany. The sample derived from a longitudinal study which tracked mono-lingual	Parents reported on children's internalising/externalising symptoms with the Child Behaviour CheckList (CBCL)	**Community-level:** Parent's also reported on children's social problems with the social problems	Longitudinal design. Growth component models were used to assess change in behaviour	Children with SLD (without ADHD) in contrast to those with ADHD, appeared to have stable behaviour over time according to parents. Children with ADHD (with and without

Author (Date)	Title	Aim of study	Population	Learning/Reading Difficulties	Context	Mental Health Concept	Third factor of interest	Methodology	Key findings
	Learning Disorders in reading and spelling from kindergarten to fifth grade	emotional and behavioural problems	126 girls) tracked to year 5, the first year of secondary school	percentile ranking in at least one domain (spelling or reading). 33% of the sample with SLD had comorbid ADHD compared to 3% in the group without SLD	German schoolchildren from kindergarten to fifth grade on their reading development and other factors		sub-scale of the CBCL	problems (internalising, externalising, attentional difficulties, social difficulties) over time	SLD), in contrast, developed more internalising/externalising behaviour over time and appeared to be especially vulnerable during times of transition such as school entrance. Regarding social difficulties, children with SLD seem to be particularly vulnerable when they first started school, and emotional/attentional difficulties peaked for these children in the last year of primary school
Hossain et al. (2021) [39]	The association between anxiety and academic performance in children with reading disorder: A longitudinal cohort study	To examine the association between overall academic performance and anxiety among children with reading disorder	One hundred and twenty-eight children and adolescents (aged 7-14 years) with reading disorder	All participants were required to have a RD to be enrolled in the schools and the study	USA. Participants were recruited from 3 schools which specialise in teaching students with reading disorder	Children's anxiety was initially measured with the School Anxiety Scale -Teacher Report (SAS-TR; Lyneham et al., 2008) but at later time-points was measured by the 8-item version of Spence's Children's Anxiety Scale (Teacher and parent versions) (SCAS; Reardon et al., 2018)	**Individual-level:** academic performance was the outcome variable in this study and was measured by teacher ratings of academic progress by survey items of the researchers own design	A 2-year longitudinal cohort study. Tracked children for three years	Children's anxiety symptoms were significantly negatively associated with their academic performance both cross-sectionally and longitudinally. Anxiety was significantly associated with reduced academic performance over time
Hossain et al. (2022) [40]	The role of grit and resilience in children with reading disorder: A longitudinal cohort study	To investigate whether grit and resilience were associated with academic performance, anxiety, depression, and quality of life among children with reading disorder	One hundred and sixty-three children and adolescents with reading disorder (6-16 years) participated	All participants were required to have an RD to be enrolled in the schools and the study	Participants were recruited from 3 schools in the San Francisco area of the USA. The schools specialised in working with students with reading disorder	Anxiety, as above. Depression – measured by the Short Mood and Feelings Questionnaire (SMFQ) and children's Quality of Life was measured by parent report with the Paediatric Quality of Life Inventory 4.0 (Varni et al., 2001). This scale assesses QOL across four domains: physical, emotional, social, and school functioning	**Individual-level**: aspects of children's coping ability, namely, grit and resilience were measured by teacher and parent report with the Grit and Resilience Scale designed by the researchers for this study's purposes. Academic performance was also an outcome variable and was	Three-year longitudinal cohort study. Repeated measures analysis using linear mixed-effects models were used to investigate associations between variables	After adjusting for age and sex, higher levels of grit and resilience were significantly related to improved mental health, academic performance, and Quality of Life among students with reading disorder

Author (Date)	Title	Aim of study	Population	Learning/Reading Difficulties	Context	Mental Health Concept	Third factor of interest	Methodology	Key findings
							measured by teacher ratings.		
Hughes & Dawson (1995)[41]	Memories of school: Adult dyslexics recall their school days	To explore schooling experiences from the retrospective perspective of adults with dyslexia	Forty-seven adults (30 males and 17 females, age range 19-51 years, Mage = 31 years) 45% were in employment, 36% were unemployed and 19% were full-time students	All participants were attending or had attended the Winchester Dyslexia Institute for tuition for at least one term	USA	The emotional experience of schooling is explored. The survey contained questions which gauged perspectives on both internalising (e.g., "I was withdrawn and shy") and externalising (e.g., "Teachers generally thought I was badly behaved") symptoms	**Individual-level:** self-esteem, shame, academic performance and the effect of being labelled "lazy". **Community-level:** Teachers' misunderstandings/misconceptions, peer problems and bullying	Mixed methods. A survey which contained both open and closed questions. Responses were analysed both quantitatively and qualitatively	The findings of this study suggested to the researcher that a cycle of poor academic performance, and being misunderstood and/or bullied by others, contributes to emotional distress amongst students with dyslexia
Ihbour et al. (2021)[42]	Mental health among students with neurodevelopmental disorders: Case of dyslexic children and adolescents	To assess self-esteem, anxiety and depression and co-morbid psychiatric conditions among Arabic speaking children and adolescents with dyslexia relative to those without dyslexia	A sample of 205 children and adolescents attending mainstream schools; 56 with dyslexia (41 boys and 15 girls Mage = 12.1 years and 149 good readers (63 boys and 86 girls, Mage = 11.71 years)	Children in the dyslexia group had been previously identified as having dyslexia. This was validated through a timed word-reading test and teacher reports	The schoolchildren were recruited from the Beni Mellal-Khenifra region of Morocco	Children/adolecents self-reported on their level of depression symptoms with the Beck-Depression Inventory (BDI; Beck et al., 1996) and their level of anxiety symptoms with Taylors Inventory of Manifest Anxiety (Taylor, 1953)	**Individual-level**: Children reported on four domains of self-esteem: general, family, social and school, with the school version of the Coopersmith Self-Esteem Inventory (SEI; Coopersmith, 1984)	Cross-sectional design. Children with and without dyslexia were compared on the psycho-social variables. This was followed by a correlational analysis of three variables (self-esteem, depression and anxiety) in each of the two groups	Children, and adolescents with dyslexia, regardless of gender, reported significantly higher levels of depression, anxiety, and poorer overall self-esteem (across all four domains). Furthermore, the researcher found significant associations between poor global self-esteem and anxiety and poor social self-esteem and depression among children/adolescents with dyslexia
Ilardi (2010) [43] Doctoral thesis	Maternal mentalization and child psychosocial adaptation for children with learning and behavioral disorders	To investigate whether mothers ability to mentalise her child's internal experience (referred to as mother's reflective capacity) is associated with their child's	The sample consisted of 18 mother/child dyads. The children were all diagnosed with or regarded as at risk for Attention-Deficit/Hyperactivity Disorder, a	Reading difficulties were determined based on children's word-reading and reading comprehension performance on sub-tests of the Wechsler Individual	USA. The children were recruited as part of a larger longitudinal study	Children's psycho-social functioning was the outcome variable and was measured by parent-report using the Child Behaviour CheckList (CBCL; Achenbach, 1991) and the Social Skills Rating System (SSRS; Gresham & Elliot, 1990)	**Family-level**: Mother's mentalisation capacity was measured through a structured interview (the PDI-2R). It is a concept that reflects the ability of mothers to reflect on their own feeling states	Mixed methods design. Mothers were interviewed about family dynamics and attitudes to their child's learning difficulties and completed standardised measures	Improved maternal mentalization was associated with higher levels of parent-reported social skills among children in this sample but was not significantly associated with children's internalising/externalising symptoms

Author (Date)	Title	Aim of study	Population	Learning/Reading Difficulties	Context	Mental Health Concept	Third factor of interest	Methodology	Key findings
		psycho-social wellbeing	Reading Disorder, or a Language Impairment and were between age 7-10 years	Achievement Test – 2nd Edition (WIAT-II)				of child behaviour	
Ingesson (2007) [44]	Growing up with dyslexia: Interviews with teenagers and young adults	To explore how "young people with dyslexia experience school in terms of wellbeing, educational achievement, self-esteem, peer relations and belief in their future" (p. 574, Ingesson, 2007)	75 adolescents and young adults (48 males, age range 14-25 years, Mage = 19 years)	All had a formal diagnosis of dyslexia adhering to DSM-4 (APA, 1995) and ICD-10 (WHO, 1992) criteria using an IQ discrepancy approach	Sweden	Measured by one interview question: "How do/did you feel in school on the whole, in terms of wellbeing?" possible answers were on a 5-point Likert scale from very bad to very good	**Individual/Community-level**: self-esteem, focusing on strengths, having more educational choice, early diagnosis, and bullying victimisation	Mixed method approach. Responses from semi-structured interviews were categorised and assigned numerical values which were then analysed via Spearman correlation	The findings of this study suggested to the researcher that the the first 6 years of schooling were most difficult for many children with dyslexia. Improved self-esteem and wellbeing among secondary school students may result from students having greater choice in school subjects and being able to compartmentalise their difficulties. Early diagnosis and focusing on strengths were constructed by the researcher as being protective of students' wellbeing
Iyanda (2021)[45]	Bullying victimization of children with mental, emotional, and developmental or behavioural (MEDB) disorders in the United States	To investigate the association between bullying victimisation and various health outcomes (including anxiety and depression) among children with a range of mental, emotional, developmental, and behavioural (MEDB) disorders	23, 494 children and adolescents via a large population-based sample (Mage = 11.65 years, 5 - 17 years) including a group classified as having a learning disorder	Determined by parent response to a survey item querying their child's MEDB status	The data was collected across 50 states within the USA	The prescence of anxiety or depression was determined by parent response to survey items	**Community-level**: bullying victimisation determined by one item on the parent survey related to their perception of their child having been bullied over the last 12 months - response format Y/N	Cross-sectional. Multivariate logistic models were used to conduct the analysis	Prevalence of bullying victimisation in the learning difficulties group (aged 6-17 years) was 11.9%. It was highest amongst adolescents with learning difficulties (14.2%) relative to younger children with learning difficulties (9.7%). Furthermore, bullying victimisation was positively associated with anxiety and depression in the sample of children with MEDB broadly

Author (Date)	Title	Aim of study	Population	Learning/Reading Difficulties	Context	Mental Health Concept	Third factor of interest	Methodology	Key findings
Jordan & Dyer (2017)[46]	Psychological wellbeing trajectories of individuals with dyslexia aged 3-11 years	To investigate age of onset and direction of effects of psychosocial difficulties in children with dyslexia in comparison to typically developing children and those with other special educational needs	The researchers used data from the UK Millennium Cohort (n = 7224 from 2003 - 2011) and tracked children from age 3-11 years	Dyslexia diagnosis was provided by teacher report	United Kingdom	Measured by teacher and parent report with the Strengths and Difficulties Questionnaire (SDQ; Goodman, 1997)	**Individual/Community-level**: Pro-sociality and Peer problems measured by teacher and parent report by the relevant sub-scales of the SDQ	Longitudinal design. Growth curve modelling controlling for age, race, gender, and family income was used to analyse the psycho-social variables by group	Onset of psycho-social difficulties among children with dyslexia appeared to occur when they commenced school suggesting to the researcher that they are secondary consequences of school experiences
Kempe et al. (2011)[47]	A longitudinal study of early reading difficulties and subsequent problem behaviors	To investigate the direction of effects between reading difficulties and a variety of "problem behaviors"	The sample consisted of 360 children recruited at kindergarten age	25% of the sample were identified as "at risk" for reading difficulties based on kindergarten word-reading scores. The groups (reading difficulty and no reading difficulty) were redefined based on reading scores at grade 3	Sweden. Children were recruited from 14 schools. All spoke Swedish as their first language	Internalising/externalising (aggression) symptoms reported by parents with the Child Behaviour CheckList (CBCL; Achenbach, 1991)	**Community-level:** children's social problems reported by parents using the relevant sub-scale of the CBCL	Longitudinal design. Children were tracked from kindergarten to grade 3 and data analysed using ANOVA with group as between-subjects factor and time as within-subjects factor	The only consistent finding across time was a positive association between reading difficulties and inattention. In this sample, reading difficulties were not associated with internalising symptoms. However, relative to parents of children without reading difficulties, parents of children with reading difficulties reported that their children experienced more social difficulties which were present even before they started school and which didn't change over time
Konstantareas & Homatidis (1989)[48]	Parental perception of learning-disabled children's adjustment problems and related stress	To investigate parental perceptions of child behaviour problems and how this is associated with parental stress	The sample consisted of 56 parents and their children (n = 28), all diagnosed with learning difficulties, 6-16 years, 20 boys and 8 girls)	All children met DSM-3 criteria for learning difficulties and were tested on a battery of tests focusing on language-related skills	Canada	Child internalising/externalising symptoms were assessed by parent report with the Child Behaviour CheckList (CBCL; Achenbach & Edelbrock, 1981)	Of interest to this study, the following **family-level factors** were measured: parental stress measured by a 5-item scale of the researchers own design; parent's locus of control (measured by the Rotter (1966) scale;	Cross-sectional	A positive association between child behavior problems and higher levels of parenting stress and lower levels of paternal self-concept was found. Maternal stress was higher among mothers who had an external locus of control.

Author (Date)	Title	Aim of study	Population	Learning/Reading Difficulties	Context	Mental Health Concept	Third factor of interest	Methodology	Key findings
							and parental self-concept measured by Coopersmith (1967) short-form, were investigated		
Kopelman-Rubin et al., (2020)[49]	The relationship between emotion regulation, school belonging, and psychosocial difficulties among adolescents with Specific Learning Disorder	To explore the influence of emotion regulation and school belonging on the psychosocial wellbeing of adolescents with a Specific Learning Disorder (SLD)	249 Hebrew speaking adolescents from 11 public schools all diagnosed with an SLD (Mage = 12.67 years, 146 boys)	SLD diagnosis met DSM-5 criteria (APA, 2013). Sub-type of learning disability was not described	Israel. Participants were recruited from a variety of schools	Was assessed by child self-report using the Total Difficulties score of the Strengths and Difficulties Questionnaire (SDQ; Goodman, 1997)	**Individual-level**: Children's emotion regulation, measured by the General Expectancy for Negative Mood Regulation Scale (Catanzara & Mearns, 1990) and **Community-level**: student's sense of school belonging measured by the Psychological Sense of School Membership Scale (Goodenow, 1993)	Cross-sectional data was analysed using Pearson correlations and a mediation model	In this study, children's emotion regulation ability was positively correlated with psychosocial wellbeing and this relationship was mediated by their sense of school belonging
La Greca & Stone, (1990)[50]	LD status and achievement: confounding variables in the study of children's social status, self-esteem, and behavioral functioning	To examine associations between academic achievement, and social and behavioural difficulties among children with learning difficulties and the influence of gender on interpersonal relationships	57 children with learning difficulties (38 male) and a comparison group of 490 without learning difficulties (233 male) all school grades 4-6. The comparison group was divided into an academic low and high achieving group. All groups matched	Learning difficulties group was based on meeting IQ-discrepancy diagnostic criteria. Sub-type of learning disability was not described	Participants were selected from four public schools located in working and middle-class neighbourhoods in Florida USA. All children with learning difficulties spent at least 50% of their school day in mainstream classes	Classroom teachers reported on child mental health using the Revised Behaviour Problem Checklist (RBPC; Quay & Peterson, 1987). This study used three of the subscales: Conduct disorder, Anxiety/Withdrawn, and attention problems	**Individual-level**: child global and social self-perception, measured by the relevant sub-scales of Harter's self-perception profile for children (SPPC; Harter, 1985). **Community-level**: "Peer liking" measured by a rating scale whereby each child was asked how much they would like to "play" with a classmate	Cross-sectional. The data from the three groups were compared on all the child and teacher ratings using two factor (Group x Sex) analysis of variance	Children with learning difficulties were given significantly lower ratings of peer liking and higher ratings of peer rejection than the other two groups. Children with learning difficulties self-reported lower social acceptance and global self-worth than the other two groups. Teachers rated girls with learning difficulties to be anxious/withdrawn. The researchers concluded that their findings suggest that academic achievement alone could not account for the variation in peer acceptance and behavioural difficulties

Author (Date)	Title	Aim of study	Population	Learning/Reading Difficulties	Context	Mental Health Concept	Third factor of interest	Methodology	Key findings
			for age, gender, race						
Lawrence et al. (2019)[51]	Effects of positive parenting on mental health in adolescents with learning difficulties	To explore the effect of family (parental mental health, family functioning and parenting practices) and adolescent social and emotional competencies on the mental health of adolescents with learning difficulties	Participants were a population sample of 24, 915 14–15-year-old adolescents (41% female, Mage = 14.47 years) and a corresponding parent	A sub-group of 5.9% of the sample were reported by parents as having a diagnosed learning disability. Sub-type of learning disability was not specified	Canada. Secondary data from the National Longitudinal Survey of Children and Youth (NLSCY) Canada	Anxious and depression symptoms (termed "adolescent distress" by the researchers) were assessed by adolescent self-report using the 7-item "behaviour scale" of the NLSCY survey. The NLSCY survey also contained a question whereby parents reported whether their child had a diagnosis of an emotional or psychological difficulty	**Individual-level:** adolescent's socio-emotional competence was measured by self-report with the Emotional Quotient Inventory-Youth Version (EQ-I; YV). **Family-level**: parents reported on family factors with survey items from the NLSCY survey (for parents) and adolescents reported on parenting style with the nurturance and monitoring subscales of the Parents and Me Scale (Lempers et al., 1989). Parental depression was measured with the short version of the Centre for Epidemiological Studies Depression Scale (CES-D scale, Radloff, 1977)	Cross-sectional. Data from one time-point of the longitudinal study were analysed. Direct and indirect effects of all variables on the outcome variable "adolescent distress" was calculated using regression analyses	Adolescent emotional socio-competence and positive parenting relationships (having nurturing parents who were aware of their activities/interests; "parental monitoring") was significantly associated with less internalising symptoms among children with learning difficulties. Parent depression had a small but significant association with adolescents' internalising symptoms, but family functioning was not found to have a meaningful association
Lawrence (2020)[52]	Bridging secondary survey data with in-depth case studies to advance understandings of youth learning	To explore psychological and relational issues among young people with learning and mental health issues	Adolescents. as above	as above	Canada. as above	Measure of youth distress (described above)	**Individual-level**:socio-emotional competence (described above) and also explored through a case study approach **Family-level**: parenting style, child-parent relationship,	Mixed methods design. A path analysis was followed by a case study approach	Findings from the quantitative section found that children's socio-emotional skills, parental mental health, and parental "monitoring" (e.g., having parents that were involved and took an interest in their activities) predicted internalising symptoms among adolescents

Author (Date)	Title	Aim of study	Population	Learning/Reading Difficulties	Context	Mental Health Concept	Third factor of interest	Methodology	Key findings
	and mental health concerns						and parental mental health (described above) **Community-level factors**: such as friendship were explored in the qualitative section of the study		with learning difficulties. Family functioning was not found to have a meaningful association. Through case study, findings support the view that parental mental health, focusing on strengths, friendships, and skills such as self-awareness and advocacy were protective. Risk factors for mental health concerns were exhaustion from school difficulties, low confidence, and avoidance
Learned (2016)[53]	"The behavior kids": examining the conflation of youth reading difficulty and behavior problem positioning among school institutional contexts	To explore young people's experience of reading difficulties within different contexts (e.g., across space and time)	Adolescents. The participants were an ethnically diverse group of 8 ninth grade high school students; their teachers (n = 8) and a group of peers identified as "proficient readers"	The participants had all received below proficient scores on district-administered standardised reading tests	USA	Behaviour "problems" from teacher and student perspectives were explored	**Community-level**: Student-teacher relationships, labelling and stigma were explored	An ethnographic qualitative approach. The researcher shadowed eight adolescents (all 14 years old) identified as "struggling readers" and interviewed them and their teachers	The analysis suggested that some teachers/school construct a "struggling reader" identity which conflates "behaviour problems" with struggling reader status; that children's "behaviour" was not a result of reading difficulties per se but rather learning environments that "position" these students as "problems". Teaching practices which focus on student's strengths and employ restorative disciplinary practices were posited to be protective of student wellbeing
Leitão et al., (2017)[54]	Exploring the impact of living with dyslexia: The perspectives of children and their parents	To explore the lived experience of children with dyslexia and their parents and investigate reasons why dyslexia might be associated with elevated risk of	13 children and adolescents (aged 10-17 years) and 21 parents (19 mothers and 2 fathers) were interviewed	All child participants had a confirmed diagnosis of dyslexia of at least one year duration	Parents were all members of the Dyslexia SPELD Foundation (DSF) in Western Australia. Child participants were all monolingual English speakers	Mental health was constructed as comprising of both internalising and externalising symptoms and was explored through a semi-structured interview process	**Individual-level**: self-esteem, the effect of receiving a diagnosis, feelings of difference **Family-level:** parent academic and emotional support. **Community-level:** social support, attitudes of others	Qualitative design. A phenomological approach using Braun and Clarke's (2006) thematic analysis method to analyse the data	Internalising rather than externalising symptoms were more commonly reported. Feeling different was an over-arching theme and highlighted broader societal challenges around notions of ability and difference. Social support (peers, parents, teachers) and the explanatory power of diagnosis were also identified as possible protective

Author (Date)	Title	Aim of study	Population	Learning/Reading Difficulties	Context	Mental Health Concept	Third factor of interest	Methodology	Key findings
		internalising and externalising symptoms					(teachers, peers), lack of government funding and teacher training, and a strengths-based approach were identified as factors which may contribute to mental health among children with reading difficulties		factors for some children's mental health
Lin et al. (2013)[55]	Reading, mathematics, and behavioral difficulties interrelate: Evidence from a cross-lagged panel design and population-based sample of US upper elementary students	To investigate the direction of effects between reading difficulties and behaviour difficulties over time and the influence of co-occurring mathematics difficulties	The researchers used data from the Early Childhood Longitudinal Study, Kindergarten Class of 1989-99 (ECLS-K), a large population-based sample of 9,324 children attending 966 public and private schools	A reading difficulties group was derived based on performance in the bottom 10% on tests of word reading and reading comprehension administered at both timepoints	USA	Internalising/externalising symptoms were measured by teacher report by a survey designed for the ECLS study	**Individual-level:** Whilst the focus of this study was to investigate the direction of effects between reading and behavioural difficulties and the influence of co-morbidity with mathematics difficulties, for the purpose of this review, we were interested in children's social and academic skills which were measured by teacher report by various sub-scales of the ECLS survey	Longitudinal design. Children were assessed at third and fifth grade	Children with reading difficulties in third grade were significantly more likely, according to teachers, to have continued reading difficulties, poor task management, poor self-control, poor interpersonal skills, and internalising symptoms in fifth grade. However, only poor task management in third grade predicted grade 5 reading difficulties
Lindeblad et al., (2016)[56]	Self-concepts and psychological wellbeing assessed by Beck Youth Inventory among pupils with	To investigate the self-image and psychological wellbeing of children/adolescents with dyslexia or severe	67 children and adolescents (aged 10-16 yrs., boys = 48, girls = 19) with reading difficulties attending compulsory school	The students were nominated by their teachers as having reading/writing difficulties. Of the total sample of 67, 21	Sweden	Symptoms of anxiety and depression were assessed by self-report with Beck's Youth Inventory (BYI; Beck et al., 2001) and compared to the Swedish norm group of 2358 children (9-18 years) provided by the BYI manual	**Individual-level**: Children's self-image (measured by self-report with the Beck Youth Inventory) and **Community-level**: attachment/attitude to	Cross-sectional design	The children with reading difficulties in this sample did not report poor self-image or symptoms of depression and/or anxiety relative to Swedish norms. 96% of students reported that they liked school, 89% reported that they felt confident to

Author (Date)	Title	Aim of study	Population	Learning/Reading Difficulties	Context	Mental Health Concept	Third factor of interest	Methodology	Key findings
	reading difficulties	reading difficulties		participants were reported to have a dyslexia diagnosis			school addressed through survey items of the authors own design		manage their schoolwork, and 75% reported that they considered reading an easy activity. The researchers speculated that these results may indicate increased teacher understanding about dyslexia and/or pedagogical approaches that suit the needs of children with reading difficulties
Lytton (1968)[57]	Some psychological and sociological characteristics of "good" and "poor achievers" (boys) in remedial reading groups: Clinical case studies"	To investigate factors that contribute to good achievement in remedial educational settings	A group of 12 children classified as "good achievers" (Mage = 8.7 years) and a group of 12 classified as "poor achievers" (Mage = 8.4 years) 8 boys and 4 girls in each group. Students were placed in each group based on their performance on a word recognition reading test and teacher nomination	All children were identified as having a reading disability and were placed in a reading-remediation class	United Kingdom	Teachers and parents reported on children's behaviour and anxiety. A composite score for anxiety for each child was based on these ratings in addition to researcher observations.	**Individual-level**: Of interest to this review were children's academic performance. Furthermore, parents completed a survey which contained items pertaining to children's developmental history (including speech development), learning/reading habits, and social skills (siblings and friends/schoolmates) and **Family-level**: family functioning (e.g., harmony in the home, acceptance of the child)	Cross-sectional. Group comparison design	Of interest to this review, the researcher found that the poor achieving group, relative to the higher achieving group, had higher anxiety, poor speech development in early childhood, and to a lesser extent, poorer family functioning
Mahdavi (2017) [58] Doctoral thesis	The influence of early parent involvement and depression on academic achievement, psychosocial behaviors, and	To investigate how early parenting variables (parent depression and involvement in school) influences academic	The study used data from the Early Childhood Longitudinal Study-Kindergarten (ECLS-K); 10, 630 children	The children were identified by their teacher as LD/RD through the ECLS-K survey	USA	Teachers reported on internalising/externalising behaviour with an adapted version of the Social Skills Rating System (SSRS; Gresham & Elliot, 1990)	**Individual-level**: Children self-reported on their motivation for learning, and teachers reported on children's academic and social performance with	Longitudinal design. Structural equation modelling	Of interest to this review, children with learning difficulties, relative to those without learning difficulties, were significantly more likely to have higher levels of internalising/externalising symptoms (teacher-reported), lower motivation to

Author (Date)	Title	Aim of study	Population	Learning/Reading Difficulties	Context	Mental Health Concept	Third factor of interest	Methodology	Key findings
	motivation in children with learning disabilities across elementary school	achievement, motivation, and psycho-social development of children (with and without learning difficulties) over time	(51% female) tracked from kindergarten to year 5. A total of 410 identified as having a learning difficulty (180 with reading difficulty and 220 with combined reading and math disability)				items from the ECLS-K survey and the adapted version of the SSRS. **Family-level**: Parents reported on their depression symptoms and involvement at their child's school through items on the ECLS-K survey		learn, and parents who reported depressive symptoms and low involvement at school. Parental depression did not appear to predict children's psychosocial wellbeing. However, findings suggest the importance of parental school involvement for children with and without learning difficulties
Martinez (2002)[59] Doctoral thesis	A comparison of learning disability subtypes in middle school: Self - concept, perceived social support, and emotional functioning	To compare the psycho-social functioning (self-esteem, social support and emotional well-being) of middle school aged students with dyslexia to those considered to be "typically achieving" and those with other types of learning disability	A sample of 120 adolescents: 6th grade (n=33), 7th grade (n=54), and 8th grade (n=33) classified as: having both reading and math disability (LD), reading disability (RD), math disability (MD) and normally achieving (NA).	Learning disability previously assessed using an IQ-achievement discrepancy definition (reading disability based on word reading or word reading and reading comprehension performance)	USA. Participants were recruited from 14 public middle schools in two large school districts in Houston, Texas.	Adolescents reported on their psycho-social functioning with the Behaviour Assessment System for Children (BASC; Reynolds & Kamphaus, 1992)	**Individual-level:** Adolescents reported on their self-esteem/self-perception with the Self-Perception Profile for Learning Disabled Students and the What I Am Like Scale (Renick & Harter, 1988**). Family/Community level:** adolescents reported on perceived levels of social support with the Social Support Scale for Children or People in My Life scale (Harter, 1985)	Cross-sectional design. Multivariate analysis of group differences	Co-morbidity of learning disabilities (but not reading or mathematics difficulties alone), was associated with more depression, lower intellectual and academic self-worth, and lower levels of perceived parent and friend support (but not teacher, class-mate or general social support) relative to adolescents without any learning difficulty
Matteucci et al., (2019) [60]	Health-related quality of life and psychological wellbeing of children with Specific Learning	To explore health-related quality of life and psychological well-being of children with	Thirty children and adoelscents (8-14 years) with a diagnosis of SLD (single or mixed type) and their	Formally diagnosed with SLD according to ICD-10 criteria (WHO, 2018)	Children and mothers were recruited in 2017 from a learning difficulties assessment and	Child mental health was measured by parent report with the Srengths and Difficulties Questionnaire (SDQ; Goodman, 1997; Tobia & Marzochi, 2017); the Test of Anxiety and Depression, parent version (TAD; Newcomer	**Family-level:** Maternal mental health measured by the EQ-5D-5L (Scalone et al., 2015), parenting stress measured by the Italian	Cross-sectional. Group comparison study	Both mothers and children with learning difficulties, compared to those without learning difficulties, reported lower levels of psycho-social wellbeing. Specifically, children with SLD had higher rates of generalised

Author (Date)	Title	Aim of study	Population	Learning/Reading Difficulties	Context	Mental Health Concept	Third factor of interest	Methodology	Key findings
	Disorders and their mothers	SLD and their mothers	mothers. Controlled for ADHD or other health conditions (unspecified) but did not specify subtypes of learning difficulty		support centre in Northern Italy. Italian was their first language	et al., 1995) and both child and parent versions of the Paediatric Quality of Life Inventory generic core scales (PedsQL; Varni et al., 2001) which provides a Psychosocial Health score	version of the Parenting Stress Index-Short Form (PSI-SF; Abidin et al., 1990; Guarino et al., 2008) and parenting self-efficacy measured by the Parenting Self-Efficacy Scale (PSE; Wittkowski et al., 2017)		anxiety, school anxiety and depression and their mothers were more likely to report symptoms of anxiety and depression. Fifty percent of mothers of children with learning difficulties reported parental distress and 80% reported a "very poor perceived ability in helping their children with school activities" (p. 51; Matteucci et al., 2019)
Maughan, B. et al. (2003) [61]	Reading problems and depressed mood	To investigate the relationship between persistent/severe reading difficulties and symptoms of depression	A community sample of 1416 children (all boys, and aged 7-10 years at the initial assessment (9.1% designated as having reading difficulties) and their families were assessed at 6-month intervals	Reading difficulties defined as California Achievement Test (CAT) percentile scores at or below 6 at recruitment and continued reading difficulties (percentile scores at or below the 50th percentile)	USA. The sample was taken from the Pittsburgh longitudinal study	Children reported on their depression symptoms from age 6 with the short version of the Mood and Feelings Questionnaire (MFQ; Angold, 1989) and parents reported on disruptive behaviour disorders among children with the Revised Diagnostic Interview schedule for Children (DISC-P, Costello et al., 1982)	Carer and/or children reported on a range of **Family-level factors** including: child-parent relationship and parenting practices (e.g., physical punishment); parental stress and coping with items from the Pittsburgh Youth Survey designed for the wider longitudinal study	Longitudinal design. Logistic regression was used to test for group differences on dichotomous variables and ordinary regressions for analysis of continuous reading scores	The researchers found robust links between reading difficulties and depressed mood among younger, not adolescent, students which could not be explained by the various family factors nor co-morbid disruptive behaviours. Rather the researchers suggest that the findings support the view of a direct association between reading difficulties and depressed mood
McArthur et al. (2022) [62]	Tracking the relations between children's reading and emotional health across time: Evidence from four large longitudinal studies	To investigate mechanisms responsible for the relationship between reading difficulties and emotional health across childhood	Used four longitudinal databases: one from the United Kingdom (n = 7,870), two from the United States (ns = 8,001 and 7,160) and one from Australia (n = 768). The researchers focused their	All included studies utilised standardised measures of word-reading and other reading difficulties as criteria	Samples derived from English speaking countries	All included studies incorporated standardised measures of internalising/externalising symptoms however measures varied across studies	In addition to attentional difficulties, modifiable **individual-level factors** of self-concept and **Community-level factors**: peer problems/bullying were investigated. Parental mental health was measured in one of the datasets, but the findings were not published	All included studies were longitudinal by design. The researchers analysed the data using Structural Equation modelling	The findings support the view that reading difficulties precede emotional and peer difficulties developmentally across childhood (and not vice versa). Specifically, reading difficulties at approximately age 7 predicted age 9 and/or 11 anxiety, depression, poor reading self-concept, bullying and other peer difficulties. In contrast, findings supported the view that behaviour difficulties (externalising

Author (Date)	Title	Aim of study	Population	Learning/Reading Difficulties	Context	Mental Health Concept	Third factor of interest	Methodology	Key findings
			analysis on data from primary school aged children				in the main section of the article and are therefore not included in this review.		behaviour) preceded later reading difficulties
Mercer (2004) [63] Doctoral thesis	Relations of self-efficacy to symptoms of depression and anxiety in adolescents with learning difficulties	To investigate relationships between self-efficacy and symptoms of depression and anxiety amongst adolescents receiving support for special education services; a subset of whom were identified as having reading difficulties	83 adolescents (13-17 years, 24 female) divided into two subsets, those with reading difficulties (n = 25) and those who met traditional aptitude-achievement discrepancy criteria for learning disability	All diagnosed as having learning disabilities according to Ministry of Education criteria. However, (n = 46) had below average performance (at or above a standard score of 80) on cognitive ability, or did not present with a discrepancy of at least 1.5 SD's between cognitive ability and academic achievement	The children were recruited from 13 public schools in British Columbia, Canada. All were receiving special education support at school. 91.6% spoke English and 95.2% were born in Canada	Symptoms of depression were measured by self-report with the second edition of the Reynolds Adolescent-Depression Scale (RADS-2; Reynolds, 2002) and anxiety symptoms with the Multi-dimensional Anxiety Scale for Children (MASC; March et al., 1997)	**Individual-level**: Children reported on their reading self-efficacy, academic self-efficacy, emotional self-efficacy, and social self-efficacy using a measure of the researchers own design. **Family and Community-level**: Children reported on their perceived levels of social support from parents, friends, and teachers using the brief version of the Social Support Questionnaire (SSQ; Sarason, 1987). Children also reported on their experience of life events (and their rating of them as negative, neutral or positive) with an adapted version of the Life Events Questionnaire (LEQ; Newcomb et al., 1981), which provides an indication of children's perception of stress,	Cross-sectional.	Children with word-reading difficulties had lower reading self-efficacy than others. However, emotional, and social self-efficacy, more adverse life experiences and lack of social support, rather than poor academic or reading self-efficacy, predicted symptoms of depression and anxiety. The researcher concluded that in this sample socio-emotional rather than academic factors were more associated with anxiety/depression symptoms

Author (Date)	Title	Aim of study	Population	Learning/Reading Difficulties	Context	Mental Health Concept	Third factor of interest	Methodology	Key findings
							according to the researchers		
Michaels & Lewandowski (1990) [64]	Psychological adjustment and family functioning of boys with learning difficulties	To investigate associations between learning difficulties, family functioning and behavioural (internalising and externalising) difficulties	The sample consisted of 59 children with learning difficulties and 65 without learning difficulties (all male and aged 6-12 years) and their parents	Previously diagnosed based on an IQ-achievement discrepancy by the Committee on Special Education within their school districts. The number with reading disability was not described	USA. Children were recruited from urban and suburban settings	Parents reported on their child's internalising and externalising symptoms with the Child Behaviour CheckList (CBCL; Achenbach & Edelbrook, 1983)	**Individual-level**: Parent's reported on their child's social competence with the social competence sub-scale of the CBCL. **Family-level**: Parent's also reported on family functioning with the Family Adaptability and Cohesion Evaluation Scale (FACES III; Olson, Portner & Lavee, 1985)	Cross-sectional. Group comparison design and ANOVA to analyse interaction between family functioning and the boys' psychosocial wellbeing	Boys with learning difficulties had higher levels of parent-reported internalising/externalising and total problem behaviour (but not aggression) relative to boys without learning difficulties. Interaction between internalising/externalising symptoms and family functioning was not significant. Nevertheless, poor family functioning in both groups was associated with lower social competence
Morgan et al. (2008)[65]	Are reading and behavior problems risk factors for each other?	To investigate the direction of effects between reading and behavioural difficulties over time	The sample included 11,515 children (50% male, average age of 65.6 months at first time-point) participating in the Early Childhood Longitudinal Study- Kindergarten Cohort (ECLS-K)	Reading scores were dichotomised based on worse 10% (reading difficulties group) or top 90% (comparison group) performance on word-reading, vocabulary and comprehension	USA	Teachers reported on internalising/externalising behaviour with an adapted version of the Social Skills Rating System (SSRS; Gresham & Elliot, 1990)	**Individual-level**: children's interpersonal skills, self-control, and study skills (e.g., task engagement) measured by teacher report using the same measure	Longitudinal design. Children were assessed in first grade and then again in third grade	Children who had reading difficulties in first grade were significantly more likely to display poor self-control, poor task engagement and higher levels of both internalising and externalising behaviours in third grade. There were no significant group differences in teacher-reported inter-personal skills
Morgan et al. (2012)[66]	Do poor readers feel angry, sad, and unpopular?	To investigate whether being poorly skilled in reading contributes to self-reported emotional and social difficulties	The sample consisted of 2, 751 children participating in the Early Childhood Longitudinal Study- Kindergarten Cohort (ECLS-K)	Children were identified as poor readers if they scored in the bottom 10% on their grade 3 ECLS-K reading test. This test measured word-reading,	USA	A NCES-modified version of the Self-Description Questionnaire-I (SDQ-I; Marsh, 1990) was used to gather children's perceptions on their internalising and externalising symptoms	**Community-level:** The version of the SDQ-I used in this study also contained a sub-scale to measure children's perceptions of peer problems	Longitudinal. Data was collected when children were in grade 3 and then again in grade 5	Children who were identified as poor readers in grade 3, relative to typically reading peers, were more likely to report higher levels of internalising (sad); externalising (angry, distractible); and social problems (lonely and unpopular) in grade 5

Author (Date)	Title	Aim of study	Population	Learning/Reading Difficulties	Context	Mental Health Concept	Third factor of interest	Methodology	Key findings
				receptive vocabulary and reading comprehension					
Newbury, et al. 2020)[67]	Cognitive, non-cognitive, and home environment correlates of reading difficulties in primary-grade students with language impairment	To investigate associations between cognitive, noncognitive (behaviour problems, social competence, effortful control), and home environment factors and reading difficulties among children with language impairment	The sample included 165 public school kindy-grade 1 children receiving intervention for language difficulties	The researchers determined a group of children with reading difficulties (36% of the sample) based on performance 1-SD below the norm on standardised tests of letter-word identification and word attack	United Kingdom. This study used data from an existing longitudinal study (The STEPS study) exploring children's speech-language therapy experiences in primary school	Children's internalising and externalising behaviour was measured by parent report using the relevant sub-scales of the Social Skills Rating Scale (SSRS; Gresham & Elliot, 1990)	**Individual-level**: children's "effortful control", and their social competence were also reported by parents with the relevant sub-scales of the SSRS. **Family-level**: "household factors", specifically, the amount of literacy-based activity that occurs in the home (measured with the HLE; Bennet et al., 2002) and family functioning (specifically the levels of "chaos and confusion" in the home) were reported by parents with the CHAOS questionnaire (Dumas et al., 2005)	Cross-sectional. Logistic regression models were used for the analysis	The researchers found that more behaviour problems and poorer social competence, but not "household factors", significantly predicted greater reading difficulties
Novita. S., (2016) [68]	Secondary symptoms of dyslexia: A comparison of self-esteem and anxiety profiles of children with and without dyslexia	To compare anxiety and self-esteem profiles in children with and without dyslexia	124 children (aged 8-11 years), 60 with dyslexia (Mage = 9.23 years) and 64 without (Mage = 8.86) matched for IQ, gender, and school grade. The control	Group membership was determined by the presence of a dyslexia diagnosis by qualified psychologists in accordance to DSM-5 (APA, 2013) and/or ICD-10	Germany. The data was collected from six schools and one clinic for children with special educational need	Anxiety was measured by child report using the Spence Children's Anxiety Scale (SCAS; Spence, 1998) which has subscales measuring 6 different types of anxiety and provides a total anxiety score	**Individual-level**: Children reported on their self- esteem with the Self-esteem test for Children and Adolescents (Schauder, 1991). This measure provides a global self-esteem score but also contains sub-	Cross-sectional data was analysed using ANOVA with the various types of self-esteem and anxiety as the dependant variables and dyslexia as the independent variable	Dyslexia was associated with higher levels of generalised (but not other types) of anxiety and low school (but not other domains) of self-esteem

Author (Date)	Title	Aim of study	Population	Learning/Reading Difficulties	Context	Mental Health Concept	Third factor of interest	Methodology	Key findings
			group had no history of learning or other specific difficulties and were recruited schools	(WHO, 2013) criteria			scales to measure school self-esteem, home self-esteem, and self-esteem during free time		
Novita et al., (2019) [69]	Children with dyslexia in different cultures: Investigation of anxiety and coping strategies of children with dyslexia in Indonesia and Germany	To investigate anxiety and coping strategies of children with and without dyslexia in two different cultural settings (Indonesia and Germany)	124 children (8-11 years) approx. 50% with a dyslexia diagnosis, 64 from Indonesia and the rest from Germany	29 of the Indonesian children and 31 of the German children had a dyslexia diagnosis. The Salzburg Test of Reading and Writing was administered during the research process (STRT; Landerl et al., 1997) to verify group membership	All German children came from 2 schools whereas all of the Indonesian children without dyslexia came from one school and those with dyslexia came from several schools in different states	Children reported on their anxiety with the Spence Children's Anxiety Scales (SCAS; Spence, 1994)	**Individual-level**: Children reported on their coping strategies with the Children's Coping Questionnaire (CCQ; Dissanayake, 2006)	Cross-sectional. Five ANOVAs by group were conducted	Children with dyslexia reported higher levels of generalised and separation anxiety but dyslexia had insignificant main effects on coping. Culture but not dyslexia status influenced children's coping strategies in this sample
Operto et al., (2021)[70]	Neuropsychological profile, emotional/behavioral problems, and parental stress in children with neurodevelopmental disorders	To investigate emotional-behavioural problems and parental stress among 3 groups of children with neurodevelopmental differences including a group with Specific Learning Disorder (SLD)	62 children (and their parents) in total; a group diagnosed with Autism Spectrum Disorder – High functioning (n = 19); ADHD (n = 21); and SLD – mixed type (n = 22, 14 males, Mage = 9.77) and a typically developing control group (males = 11,	All children in the SLD group were formally diagnosed by two experienced neuropsychologists according to DSM-5 criteria (APA, 2013)	The clinical samples were recruited via the Child and Adolescent Neuropsychiatry Unit at the University Hospital of Salerno (Italy)	Children's internalising/externalising symptoms were measured by parent report on the Children's Behaviour CheckList (CBCL; Achenbach & Rescorla, 2001)	**Family-level**: parental stress was measured by the Parenting Stress Index Short-Form (PSI-SF; Abidin, 1995)	Cross-sectional. Group differences were assessed using Kruskall-Wallis Ttests and U-Mann Whitney tests	Parental stress and child mental health concerns were higher across all three of the disability groups compared to controls

Author (Date)	Title	Aim of study	Population	Learning/Reading Difficulties	Context	Mental Health Concept	Third factor of interest	Methodology	Key findings
			Mage = 10.88 years)						
Palti (1998) [71] Doctoral thesis	A study of the socio-emotional aspects of educationally resilient dyslexic students	To investigate socio-emotive factors relating to educational resilience among children with dyslexia	The sample consisted of eighty children (7-11 years, all boys) with dyslexia	All the boys in the sample were formally diagnosed with dyslexia following parent/teacher concern and using the ability-achievement definition of dyslexia	United Kingdom	Parents reported on children's social, emotional, and behavioural functioning using a single open-box item on a questionnaire of the researchers own design. The teacher questionnaire had a section where the teacher can tick whether they perceived the child to experience certain internalising (e.g., anxious) or externalising (e.g., aggression) behaviour	**Individual-level**: Children's educational resilience was measured using standardised tests of literacy skills. Children also reported on their self-esteem by self-report with the BIG Steem Questionnaire (Maines and Robinson, 1988).	Mixed methods and group comparison study. Boys with dyslexia who performed better academically (referred to as "academically resilient") were compared on socio-emotional variables to those who were performing less well academically	The educationally resilient boys with dyslexia reported higher levels self-esteem but more socio-emotional difficulties compared to those categorised as less educationally resilient. From this, the researcher suggested that boys with dyslexia who are educationally resilient may "pay an emotional cost for their academic success" (p.127)
Panicker & Chelliah (2016)[72]	Resilience and stress in children and adolescents with Specific Learning Disability	To assess the level of stress, resilience, depression and anxiety among two groups of children; those who have Specific Learning Disability (SLD) and those who have Borderline Intellectual Functioning (BIF)	The sample consisted of children and adolescents (age range 7-17 years, Mage = 12.15 years, 92% male); 41 diagnosed as having an SLD; and a comparison group of 41 diagnosed as having borderline (IQ 75-79) intellectual functioning. Psychiatric co-morbidity was present in 41% of the sample with SLD (13% had co-	Diagnosis for SLD was assessed by a qualified clinical psychologist based on performance two standard deviations below their current academic year at school in spite of having normal intellectual functioning. Twenty seven percent of the sample of children/adolescents with SLD had reading disorder	India. Ninety eight percent of the children in the SLD group were attending mainstream schools	Depression, anxiety, and stress were measured by the 42-item self-report Depression, Anxiety and Stress Scale (DASS; Lovibond & Lovibond, 1995)	**Individual-level**: Children's levels of stress (measured by the DASS) and resilience (measured by the Resilience Scale-14 (RS-14; Wagnild, 2009) which measures an individual's capacity to manage life challenges and stressors. **Family-level**: parent's level of awareness about their child's learning difficulties was measured through an interview schedule of the researchers own design	Mixed methods design	75% of students with SLD had low level of resilience and 51% were "reprimanded" by their parents for not performing well academically even though 91% of parents were aware that their child had an SLD. Parents reported low levels of SLD specific training. However, these parental factors were not associated with children's self-reported levels of resilience, stress, anxiety and depression. In this sample, the SLD group reported higher levels of anxiety relative to the BIF group and children with reading difficulties had statistically significant higher levels of anxiety than those without reading difficulties. Of those with SLD, 14.2% reported severe depression;

Author (Date)	Title	Aim of study	Population	Learning/Reading Difficulties	Context	Mental Health Concept	Third factor of interest	Methodology	Key findings
			morbid ADHD; 3% with depression and 2% with anxiety) and 27% with BIF						23.8% of children reported severe anxiety; and 16.6% reported severe stress
Parhiala et al. (2015)[73]	Psychosocial functioning of children with and without dyslexia: A follow-up study from ages four to nine	To investigate the psycho-social functioning of children with dyslexia before and after the transition to primary school.	Tracked the development of 170 children (half at risk of reading difficulties and half not) over the transition from home to school.	After grade 2 a dyslexia group (n = 39 children, 20 girls and 19 boys) was formed. They fulfilled diagnostic criteria for dyslexia using standardised tests of word-reading difficulty	Finland	Internalising and externalising symptoms were measured by parent report using the relevant sub-scales of the Behaviour Assessment for Children (BASC; Reynolds & Kamphaus, 1992)	**Individual-level:** Children's adaptability and social skills were measured by parent report using the relevant sub-scale of the BASC	Longitudinal design	Children with dyslexia, relative to those without dyslexia, were rated by parents as having poorer social skills and attentional difficulties but no link between dyslexia and internalising/externalising symptoms was found in this sample
Pih (1984) [74] Doctoral thesis	A comparison of normal and disabled readers in elementary school on intellectual, self-esteem, and anxiety factors	To investigate associations between reading, cognitive, and non-cognitive (anxiety/self-esteem) factors	The sample consisted of 40 children (aged 7-8, grade 2-3) identified as poor readers and 40 typically reading peers matched by age, gender, grade, race, and IQ	The groups were determined by children' scores on a standardised reading test (word reading and comprehension ability). Further testing of various aspects of reading were conducted to ascertain "reading level" which was treated as a correlational variable in the second part of the study	USA. The reading difficulties group were all accessing reading remediation classes	Children's anxiety was measured by self-report with the Revised Children's Manifest Anxiety Scale (RCMAS; Reynolds & Richmond, 1978)	**Individual-level**: Children's self-esteem was measured by self-report with the Coopersmith Self-Esteem Inventory (SEI; Coopersmith, 1984)	Cross-sectional. ANOVA and correlational analysis	Typical readers, compared to those identified as poor readers, scored significantly higher on self-esteem but no group differences in anxiety were reported. There was a significant positive relationship between self-esteem and reading achievement in the typical reading group and a significant negative relationship between anxiety and reading achievement in the reading difficulties group

Author (Date)	Title	Aim of study	Population	Learning/Reading Difficulties	Context	Mental Health Concept	Third factor of interest	Methodology	Key findings
Russell et al. (2015)[75]	Behavioural difficulties that co-occur with specific word reading difficulties: A UK population-based cohort study	To investigate the relationship between word-reading difficulties at age 7 and a range of social, emotional, and behavioural difficulties at age 11	Utilised the Millennium Cohort Study (MCS) data from 18519 participating families	13, 423 children were tested at age 7 for word reading ability with the British Ability Scales (BAS; Elliot et al., 1996). A reading difficulties group was formed based on performance 2 SD below chronological age norms	United Kingdom	Measured by teacher and parent report with the Strengths and Difficulties Questionnaire (SDQ; Goodman, 1997)	**Individual/Community-level**: children's pro-sociality and peer problems both measured by parents and teachers via the relevant subscales of the Strengths and Difficulties Questionnaire (SDQ; Goodman, 1997)	Longitudinal design. Tracked children from age 7 until 11 years	In this sample, word-reading difficulties were associated with (and appeared to precede) elevated levels of emotional difficulties, peer problems, conduct problems and hyperactivity/attentional difficulties measured by teacher and parent report
Sahin et al., (2018)[76]	The relationship of clinical symptoms with social cognition in children diagnosed with attention deficit hyperactivity disorder, specific learning disorder or autism spectrum disorder	To study Theory of Mind (ToM) among children with Specific Learning Disorder (SLD) in comparison to children with other neurodevelopmental differences and those considered typically developing	The sample consisted of children aged 7-12 years: 24 children diagnosed with Specific Learning Disorder (SLD); 24 with ADHD; 26 with autism; and 24 with no known neurodevelopmental difference	Diagnoses conformed to DSM-5 criteria. Additionally, co-morbidities were exclusion criteria for group membership in this study	Turkey. The children with neurodevelopmental differences were recruited from a clinical context and the typically developing controls were all volunteer relatives of health workers.	Internalising/externalising symptoms were measured by parent and teacher report with the Aberrant Behaviour Checklist (ABC) (Aman et al., 1987) and the Turgay DSM-4 Disruptive Behaviour Disorders Rating Scale - Teacher form (Turgay, 1994)	**Individual-level**: The variable of interest to this review is children's social skills which were reported on by parents using the Social Responsiveness Scale (SRS) however the researchers also measured children's Theory of Mind (TOM) skills	Cross-sectional design	Children with neurodevelopmental disorders (including those with SLD) had deficits in theory of mind, relative to typically developing controls, independent of language skills and intelligence. Furthermore, the researchers found a significant correlation between poor social cognition skills and poorer social skills, learning, attention, and behaviour
Sainio et al. (2019)[77]	The role of learning difficulties in adolescents' academic emotions and academic achievement	To examine associations between learning difficulties, academic emotions, and academic achievement	The sample consisted of 845 grade 6 children (455 girls, 390 boys)	A reading difficulties group was determined by performance 1 standard deviation below the mean of the whole sample on standardised tests of: word	The study was part of a broader longitudinal study following a community sample of Finnish students across the transition from	Children reported on their academic emotions with the Finnish version of the Achievement Emotions Questionnaire (AEQ; Pekrun et al., 2011). The researchers focused on "hope" (e.g., "I have an optimistic view towards studying"), enjoyment (e.g., "I enjoy acquiring new knowledge"), and anxiety (e.g.,	**Individual-level**: Children self-reported on their overall academic achievement; literacy achievement (grade in literacy) and math achievement (grade in mathematics); and	Longitudinal. Data from the fall and spring of grade 6 was analysed. Gender, difficulty in other academic areas, and depression were controlled in the analysis.	The results of interest to this review are that students with reading difficulties reported lower hope and anxiety towards reading than those without reading difficulties. Furthermore, student's hope played a mediating role between learning difficulties and academic (mathematics and literacy)

Author (Date)	Title	Aim of study	Population	Learning/Reading Difficulties	Context	Mental Health Concept	Third factor of interest	Methodology	Key findings
				identification, decoding, and reading fluency	primary to secondary school.	"I get tense and nervous while studying")	attitude to their learning (hope and enjoyment – described under MH concept)		achievement. The researchers concluded that subject-specific academic emotions should be considered in regard to the academic achievement of students with learning difficulties
Singer (2005) [78]	The strategies adopted by Dutch children with dyslexia to maintain their self-esteem when teased at school	Through structured interviews with children the researcher aimed to explore their school experiences, specifically experiences of being teased, coping with teasing, and their psychological well-being (self-esteem and mental health)	The sample consisted of 60 Dutch children with dyslexia (aged 9-12 years) in mainstream class settings	All had been formally diagnosed with a primary diagnosis of dyslexia of at least one year duration, with at least average IQ and no other known psychopathology	Netherlands. The children were recruited via educational guidance services providing remedial support to children with learning difficulties. 91% of the sample were ported to be receiving extra remedial help from school and/or out of school service providers	The researcher explored children's psycho-social functioning broadly with a focus on self-esteem	**Individual-level**: Children's self-esteem, emotion regulation and coping strategies, and **Family/Community level**: perceptions of social support and peer difficulties/bullying were explored in this study	Mixed methods design. All children were invited to respond to fictitious scenarios involving the school experiences of children with dyslexia. After this, children were invited to discuss their own experiences, and 85% of the children did this via a semi-structured interview	The findings suggested that many children of this age feel embarrassed about their dyslexia and vulnerable to negative peer (self) comparisons. The author concluded that dyslexia directly affects children's self-esteem and makes them vulnerable to teasing. Further, that withdrawn or disruptive behaviour may be serving a protective function for children (in terms of addressing bullying); and that parents are the most important source of emotional and practical support
Singer (2007) [79]	Coping with academic failure, a study of Dutch children with dyslexia	A companion piece to the study above, in this study the researcher aimed to explore how children with dyslexia cope with repeated academic failure and how this relates to their self-esteem and mental health	9–12-year-old children (as above)	as above	Netherlands. as above	The researcher explored children's psycho-social functioning broadly with a focus on self-esteem	**Individual-level**: Children's self-esteem and coping strategies were explored and **Family/Community level:** perceptions of social support and peer difficulties/peer comparisons/bullying were explored in this study	Mixed methods design. All children were invited to respond to fictitious scenarios involving the school experiences of children with dyslexia. After this, children were invited to discuss their own experiences, and 56 children did so.	The researcher found that parent support was crucial for children's wellbeing. In contrast, peers were more likely perceived as threats to self-esteem. The protective function of adult role models (generally family members) was explored and the negative influence of bullying. Children were described as being motivated to protect their self-esteem and employed different approaches to do so such as: "1) working hard and committing to standards, 2)

Author (Date)	Title	Aim of study	Population	Learning/Reading Difficulties	Context	Mental Health Concept	Third factor of interest	Methodology	Key findings
									lowering standards 3) seeking support from significant others (i.e parents and teachers), and, 4) avoiding comparisons with others (i.e. peers)" (p. 314)
Smart et al., (2019) [80]	Social mediators of relationships between childhood reading difficulties, behaviour problems, and secondary school non-completion	To examine whether key indicators of social connectedness mediate relationships between childhood reading difficulties/behavioural problems and secondary school non-completion	Four groups of children were formed by the researchers for this study: those with reading disorders (n = 38, 37% male); behavioural difficulties (n = 66, 58% male); both reading difficulties and behavioural difficulties (n = 38, 66% male); and neither reading difficulties or behavioural difficulties (n = 125, 35% male)	Children who performed in the lowest 16% on a standardised measure of word-reading were classified as those with reading difficulty	Australia. Data was drawn from the Australian Temperament Project an Australian longitudinal study that has followed a community sample of 2,443 children and their families from infancy to young adulthood	Internalising/Externalising symptoms were measured by parent and teacher report with the Rutter Child Behaviour Questionnaire (CBQ; Rutter, 1967; Rutter et al., 1970)	**Family/Community-level factors:** Children reported on their family and peer attachment with the Inventory of Parent and Peer Attachment (IPPA; Armsden & Greenburg, 1987) and both parents and children reported on various school factors such as: teacher attachment and approach to learning such as school engagement (including attendance, diligence), and school involvement (parent and child) with the ATP- devised School Problems Scale (SPS; Prior et al., 2000)	Longitudinal design. The analysis used data from the 6th (7-8 years), 9th (12-13 years), 10th (13-14 years), and 13th (19-20 years) time-points	The group with reading difficulties (without behavioural problems) reported more difficulty with understanding their schoolwork in the high school context, relative to children without reading/behavioural difficulties but were similar in terms of other measures. In contrast, children with behavioural problems (with and without co-occuring reading difficulties) reported significantly poorer family and school attachment/involvement
Snowling et al., (2007)[81]	Children at family risk of dyslexia: A follow-up in early adolescence	Using a longitudinal design, the researchers aimed to investigate a range of genetic and environmental risk factors for psycho-social outcomes	Fifty children with a family history of dyslexia and a control group of 20 without a family history of dyslexia were followed from infancy until	At time-point 4 (when the children were aged 12-13 years) 42% of children (11 boys and 10 girls) who had been previously classified as at family risk for	United Kingdom. This study represented the fourth phase of a longitudinal study which tracked British children at risk of literacy	Parents reported on children's internalising/externalising symptoms with the Strengths and Difficulties Questionnaire (SDQ; Goodman, 1997)	**Individual-level**: Children reported on their self-perception in three domains (social, academic, and physical competence) using Harter's Self-perception Profile for Children (Harter, 1985)	Mixed methods. Three groups (at risk "impaired"; at risk "unimpaired" and control group) were compared on the language, literacy, and SDQ factors	There was a significant positive association between literacy (reading and spelling) difficulties, attentional difficulties, and parental ratings of hyperactivity (but not conduct problems) and emotional difficulties, poor self-ratings of scholastic competence but not overall self-esteem or peer problems. There was also

Author (Date)	Title	Aim of study	Population	Learning/Reading Difficulties	Context	Mental Health Concept	Third factor of interest	Methodology	Key findings
		among children identified in early childhood as being at risk of literacy difficulties	early adolescence (time-point 4). This study analysed the time-point 4 data	dyslexia were classified as having literacy difficulties based on reading and spelling attainment 1 standard deviation below the mean of the control group. This group (at risk impaired) were compared on a range of indicators to children who were at family risk for dyslexia but did not develop literacy difficulties (at risk unimpaired) and the original control group of children	difficulties from infancy		in addition to their print exposure using a questionnaire that tapped recognition of author names, book titles etc. Children were also assessed for spoken language skills. **Family/Community-level:** parents reported on children's peer problems with the relevant sub-scale of the SDQ. A parent interview gleaned information relating to a wide range of family (e.g., family stress and family literacy environment) and school (e.g., home-school liaison) factors	(externalising, internalising, peer problems), self-perception (scholastic, social acceptance and athletic), family environment and educational factors measured at time-point 4	evidence of a negative association between literacy difficulties and maternal well-being (higher levels of maternal stress and depression), family life, and parental worries about their child's progress at school even though most children were receiving support at school
Sorrenti (2019)[82]	The predicting role of school learned helplessness in internalizing and externalizing problems: An exploratory study in students with Specific Learning Disorder	To investigate learned helplessness as a predictor of the development of internalising/externalising difficulties in children with learning difficulties	The sample consisted of 84 children and adolescents (ranged from first grade to upper secondary school; Mage = 12.9 years, 52 males, 44 with learning difficulties and 40 without learning difficulties). The number of	All children in the learning difficulties group had an SLD diagnosis which conformed to DSM-5 criteria. Further, the researchers administered standardised tests in reading and writing to ensure that the SLD group met	All children were attending school in Messini, Italy	Internalising/externalising symptoms were measured by self and parent report with the relevant versions of the Child Behaviour CheckList (CBCL; Achenbach, 1991)	**Individual-level**: Children reported on their learned helplessness in school using the learned helplessness sub-scale of the Italian version of the Learned Helplessness Questionnaire (LHQ; Sorrenti et al., 2015). In this study learned helplessness referred to student's passiveness and lack	Cross-sectional	Children with learning difficulties, relative to those without learning difficulties, had higher levels of internalising symptoms. Furthermore, learned helplessness was a predictor of internalising and externalizing symptoms in children with, but not without, learning difficulties

Author (Date)	Title	Aim of study	Population	Learning/Reading Difficulties	Context	Mental Health Concept	Third factor of interest	Methodology	Key findings
			children with dyslexia was not described	diagnostic criteria			of confidence regards academic tasks		
Speers (2019)[83] Doctoral thesis	An auto/biographical investigation exploring the life-stories of adults with dyslexia	To explore individual's experiences with dyslexia over the lifespan including in education and employment settings	Eight adults with a formal diagnosis of dyslexia including the researcher participated in the study	All participants self-identified as having dyslexia	United Kingdom	Childhood school behaviour in association with dyslexia were explored	**Individual-level**: Identity issues and the value of early diagnosis, self-esteem, shame (embarrassment), concealment and disclosure were explored by the participants. **Family-level**: the importance of parental support and understanding. **Community-level:** supportive learning environments, finding strengths	Qualitative design. Described as an autobiographical narrative inquiry. The researcher developed themes from the interview transcripts	Childhood externalising rather than internalising behaviour was highlighted. This behavior appeared to be especially related to the school context and dyslexia-related school difficulties such as being placed in "low" groups for learning and having difficulties with learning (leading to frustration). Early diagnosis and parental (especially maternal) support appeared to be protective of self-esteem. Participants described feeling ashamed of their learning difficulties and various coping strategies such as withdrawing and trying to conceal their differences at school and beyond
Stanley et al. (1997)[84]	Differences in depression and self-esteem reported by learning disabled and behavior disordered middle school students	To investigate self-reported group differences in self-esteem and depression among two at risk groups of students	Sixty-one middle school students (age and gender not provided but middle school students are typically adolescents) 33 classified as having learning disability and 28 as having behaviour disorder	The students had been classified as having a learning disability. The number with dyslexia was not specified	no context provided, believed to be USA	Depression measured by self-report with the Children's Depression Inventory (CDI; Kovacs, 1992)	**Individual-level**: self-esteem measured by children's self-report on the Coopersmith Self-esteem Inventory (CSEI; Coppersmith, 1990)	Cross-sectional	The levels of depression between the two groups (learning disabled vs behaviourally disordered) were similar. At t the "mildly depressed" level, the researcher felt the levels of depression were higher than what would be expected among children generally. Of interest to this review, the children with learning difficulties reported significantly lower levels of self-esteem than did those classified as having a behaviour disorder
Sukovieff & Kruk (2021) [85]	Reading difficulty and socio-emotional	To investigate the effect of "age of	Forty-five children (ages 9-12 years) with	Reading difficulties initially determined by	Winnepeg, Canada	Children reported on their internalising/externalising symptoms with the modified version of the	**Individual-level**: children's ability to cope with their	Cross-sectional. Mediational analysis using the	Early identified children (before grade 1) were more likely to report increased externalising and

Author (Date)	Title	Aim of study	Population	Learning/Reading Difficulties	Context	Mental Health Concept	Third factor of interest	Methodology	Key findings
	adjustment: Internalizing patterns depend on age of identification	identification" on children's coping strategies and internalising/externalising symptoms	reading difficulties were recruited from schools and childcare centres	teacher/parent nomination and then confirmed through scores on standardised tests of word-reading administered by the researchers		Self-Description Questionnaire (SDQ; Marsh, 1990) used by other studies using the Early Childhood Longitudinal Study; this scale has six items relating to externalising symptoms and eight items relating to internalising symptoms	reading difficulties was measured by self-report using an adaptation of the Response to Stress Questionnaire-School version (RSQ; Connor-Smith et al., 2000) - items were adapted to refer to school/reading related stress. Additionally, the age of identification of the child's reading difficulties was reported by an item on the parent survey and coded dichotomously as early (poor reading identified ≤ 6 years) and late identified (reading difficulties identified between 7-12 years)	PROCESS macro (Hayes, 2018).	internalising symptoms relative to those classified as being identified later. Furthermore, the way children reported coping with their reading difficulties did not appear to influence the association between age of identification and mental health outcomes
Terras et al., (2009) [86]	Dyslexia and psycho-social functioning: An exploratory study of the role of self-esteem and understanding	To investigate the nature of the relationship between self-esteem, mental health, and family factors (i.e., understanding dyslexia and the parent-child relationship)	A convenience sample of 68 children and adolescents with dyslexia (age range = 7.67–16.58 years, Mage = 11.2 years, 44 male) and their parents were recruited through a dyslexia service/advocacy	All children had been assessed as having dyslexia by an educational psychologist using the IQ-reading ability discrepancy approach	United Kingdom. All families were members of "Dyslexia Action" in Scotland. High percentage of parents (52%) were in professional/managerial occupations	Children's socio-emotional difficulties were measured by parent report with the Strengths and Difficulties Questionnaire (SDQ; Goodman, 1997). A sub-sample of 35 children completed the child version of the SDQ	**Individual-level**: Children's self-esteem was measured by child and parent report with Harter's Self-profile for Children (SPCC; Harter, 1985) and survey items measured self-understanding of dyslexia. **Family-level**: Parent understanding of dyslexia and parent/child relationship quality	Mixed methods design. Cross sectional data was analysed by Pearson correlations and compared to population norms	Dyslexia was associated with socio-emotional difficulties. Low self-esteem positively correlated with socio-emotional difficulties in this sample. Further, there was a positive association between poor academic self-esteem and SDQ "emotional difficulties". Children's socio-emotional wellbeing was associated with having more positive attitudes to dyslexia (both parents and children) and more positive peer and parent relationships

Author (Date)	Title	Aim of study	Population	Learning/Reading Difficulties	Context	Mental Health Concept	Third factor of interest	Methodology	Key findings
			group in Scotland				was measured by survey items of the researcher's own design. **Community-level:** Peer problems were measured by parents using the peer problem subscale of the SDQ		
Thomson et al. (1990)[87]	Behavior problems in children with the presenting problem of poor school performance	Compared behavioural difficulties among a variety of children with low school performance	A total sample of 79 children and adolescents (70% males, aged 6-15 years) divided into a learning disabled group; a group with low IQ; a group with borderline IQ; and a control group with normal IQ and no learning difficulties	Thirty-four children met criteria for learning disability based on IQ-discrepancy and performance in the lowest 20% of children on standardised tests of reading, writing or mathematics. The number of children with dyslexia was not reported	USA. All children were experiencing low school performance	Internalising/externalising symptoms were assessed by parent report using a combined checklist based on items from two standardised measures (one being the Child Behaaviour CheckList (Achenbach & Edelbrock, 1983) and the other being the Missouri Children's Behaviour Checklist (MCBC; Sines, Pauker, Sines, & Owen, 1969) which contains six subscales which purport to measure aggression, inhibition, activity level, sleep disturbance, somatization, and sociability	**Individual-level**: Children reported on their self-esteem using Harter's Perceived Competency Scale for Children (Harter, 1982) which includes sub-scales that relate to cognitive, social and physical self-worth.. Parents reported on children's sociability with the MCBC. F**amily-level**: family functioning was measured by parent-report with the Family Relationship Index (FRI; Holahan & Moos, 1981) a measure of family cohesion, expressiveness and conflict	Cross-sectional. ANOVA and MANOVA were used to analyse the data	The MCBC sociability factor did not relate to internalising/externalising symptoms in this study. Nevertheless, across subgroups, more behavioural difficulties were associated with lower self-worth and family functioning described as being more controlling and less supportive. Further, internalising symptoms were negatively related to lower levels of social self-worth
Tistan (1993)[88] Doctoral thesis	Learning disabilities and depression in children: The development of self-concept	To investigate the association between learning disabilities depression, self-esteem, and	The sample consisted of 90, 8-11 year old children (60 with learning difficulties and 30	The children with learning difficulties were recruited from special education classes within	Described by the researcher as a middle-class suburban community with minimal ethnic diversity.	Children's symptoms of depression were measured by self-report with the Children's Depression Inventory (Kovacs, 1983)	**Individual-level**: children's self-concept was measured by self-report with the Piers-Harris Self-Concept Scale (Harris, 1969) and their	Cross-sectional. Described by the researcher as a quasi-experimental group comparison design	Children with learning difficulties, compared to those without learning difficulties, reported more depression in this study. Furthermore, poor self-concept and poor reading comprehension were associated with more

Author (Date)	Title	Aim of study	Population	Learning/Reading Difficulties	Context	Mental Health Concept	Third factor of interest	Methodology	Key findings
	and achievement	school achievement	without learning difficulties)	mainstream school settings and had learning difficulties confirmed through psycho-educational assessment	New Jersey, USA		reading achievement (as a measure of school achievement) was measured by the Woodcock Reading Mastery Tests – Revised (Woodcock, 1987)		depression among children with learning difficulties. The researcher suggested that the findings support a view that that learning difficulties in combination with depression may be associated with poor self-concept rather than learning disabilities alone
Törő et al., (2018)[89]	Reading disability spectrum: Early and late recognition, subthreshold, and full comorbidity	To investigate the comorbidity of reading difficulties with subthreshold and full psychiatric disorders and the influence of age of reading difficulties recognition on this relationship	130 children and adolescents (7-18 years) classified as those with reading difficulties (n = 48, Mage = 10.23 years, 40% girls) and those without reading difficulties (n = 82, Mage=9.66 years, 39% girls)	A Hungarian adaptation of the Dyslexia Differential diagnosis Maastricht-Hungarian Standard Test (3DM-H; Toth et al., 2014) was used to verify group membership	Children were recruited through the Department of Children and Family Services, Vecses City Local Government, Hungary.	The Mini International Neuropsychiatric Interview Kid, a diagnostic interview which assesses for 25 child psychiatric disorders related to the DSM-Fourth edition (APA, 1994) was used. This produces a total externalising and total internalising score per participant. Children < 13 years were interviewed with a parent and children ≥ 13 years were interviewed alone	**Individual-level**: Age of recognition of reading difficulties were investigated in association with the severity of reading difficulties and mental health concerns	Cross-sectional data	Higher levels of internalising and externalising disorders were more prevalent in the group with, relative to those without, reading difficulties, regardless of reading difficulty severity. When considering both sub-threshold and full psychiatric diagnoses together, externalising symptoms and mood disorders were more prevalent among children whose reding difficulties were recognized late, rather than early in their life
Tsovili (2004)[90]	The relationship between language teachers' attitudes and the state-trait anxiety of adolescents with dyslexia	To investigate anxiety among adolescents with dyslexia and the role of teacher attitudes	68 adolescents with dyslexia (52 male; Mage = 15 years) and their language teachers (31 males) selected at random from a hospital outpatient service and a specialist dyslexia centre	All child participants had been previously diagnosed with dyslexia by a multidisciplinary team within a hospital setting	Greater Athens area, Greece	The adolescent's state-trait anxiety was measured by a Greek adaptation of the State-Trait Anxiety Inventory for Children (Psychountaki, 1995; Spielberger et al., 1973). The state anxiety component of the measure was adapted by the researchers to measure reading-related anxiety	**Individual-level**: through interviews the researcher explored students' attitudes to learning and ways of coping with difficulties. **Community-level:** Teachers completed a questionnaire of the researchers own making (called the Teacher Questionnaire) which purported to measure student-teacher relationship and	Mixed methods design. Group differences on variables of interest were first computed followed by correlational and regression analysis. The adolescents with the lowest and highest levels of reading anxiety (5% of research group at both ends = 9	Adolescents with dyslexia reported higher reading anxiety but lower levels of trait anxiety than those without reading difficulties. During the interviews, problem-focused coping styles and teacher understanding were identified as protective whereas "learned helplessness" was described as contributing to risk for reading anxiety

Author (Date)	Title	Aim of study	Population	Learning/Reading Difficulties	Context	Mental Health Concept	Third factor of interest	Methodology	Key findings
							teaching style/behaviour/attitude	participants) were then interviewed	
Turunen et al. (2019)[91]	Word reading skills and externalizing and internalizing problems from grade 1 to grade 2 - Developmental trajectories and bullying involvement in grade 3	To investigate associations between word-reading skills and internalising/externalising behaviour, on bullying involvement over time	A sample of 480 Finnish children (Mage = 7 years 2 months at start of study) were tracked from kindigarten to year 3	Groups of below average and skilled readers were formed based on performance on word-reading tests administered at all time-points during the study	Finland	Externalising and internalising symptoms were evaluated by teachers at all three time-points using the Strengths and Difficulties Questionnaire (SDQ; Goodman, 1997)	**Community-level**: Grade 3 children reported on their bullying involvement (with sub-scales measuring: bullies /victims/ and bully-victims) with the Olweus Bully/Victim Questionnaire (Olweus, 1996).	Longitudinal design	The researchers concluded that the findings suggest that reading difficulties alone do not increase the risk of bullying involvement but add cumulative risk, alongside internalising/externalising symptoms, to children being bullying perpetrators or bully-victims
Undheim et al. (2008)[92]	Psychosocial factors and reading difficulties: Students with reading difficulties drawn from a representative population sample	To investigate a variety of psycho-social variables in a community-based sample of adolescents with and without reading difficulties.	A sample of 2,464 Norwegian adolescents (12-15 years, 50.8% girls) participated. The sample contained a sub-sample with reading difficulties (n = 199)	Determined by two self-report items: "Have you had specific reading and writing problems (dyslexia) in the last 12 months?" and are the amount of reading problems "large problems" "some problems" or "no problems"	Norway	Depression was measured by self-report with the Mood and Feelings Questionnaire (MFQ; Angold, 1989)	**Individual-level:** Children reported on their self-esteem with the Self-Perception Profile for Adolescents (SPPA; Harter, 1988, Wichstrom, 1995); their levels of school stress with the Early Adolescent Stress Questionnaire (EASQ; Sund et al., 2003) **Family and Community-level:** Children reported on their attachment to parents and peers with the Inventory of Parent and Peer Attachment (IPPA; Armsden & Greenberg, 1987)	Cross-sectional. A series of regressions was conducted to examine differences between the two groups (with and without reading difficulties) on the psycho-social variables	Adolescents with reading difficulties, compared to those without reading difficulties, reported lower attachment to parents (but not peers), lower global self-worth as well as higher rates of depression, school stress, and worry about going to school. Adolescents' self-esteem did not moderate the association between school stress and reading difficulties in this sample

Author (Date)	Title	Aim of study	Population	Learning/Reading Difficulties	Context	Mental Health Concept	Third factor of interest	Methodology	Key findings
Undheim et al. (2011)[93]	Emotional and behavioral problems among school adolescents with and without reading difficulties as measured by the Youth Self-Report: A one-year follow up study	To investigate the association between reading difficulties and emotional/behavioural difficulties across time	A sample of 191 Norwegian adolescents, 12-15 years, 7.8% with reading difficulties	Based on adolescent's self-report of reading difficulties at Time-point 1	Norway	Internalising/externalising symptoms measured by self-report with the Norwegian version of the Youth Self Report - Child Behaviour CheckList (YSR-CBCL; Achenbach, 1991)	**Community-level**: Adolescent's social difficulties/experiences was measured by self-report with the 8-item social problems sub-scale of the YSR-CBCL	Longitudinal design.	Reading difficulties were associated with higher self-reported internalising symptoms, externalising symptoms, and social difficulties. Furthermore, reading difficulties predicted high levels of social difficulties prospectively irrespective of educational placement
Valenti (1986)[94] Doctoral thesis	Manifest aggression, state/trait anxiety, locus of control, perceived parental attention, and reading level among learning disabled adolescent males	To investigate family and personality factors in association with anxiety and aggression among adolescent males with reading/learning difficulties	49 adolescents (aged 12-18 years, all male)	All had met an IQ-discrepancy diagnostic criteria for learning disability. Reading level was also assessed by the researcher by a standardised reading test	Boston, USA. The boys were recruited from specialist schools for children with learning difficulties	Aggression (externalising) and State/Trait anxiety (internalising) were measured by child self-report with the Manifest Aggression Scale (Jesness, 1966) and The State-Trait Manifest Anxiety Scale (version not described)	**Individual-level**: Children reported on their locus of control with the Locus of Control Scale (LOC; Nowicki-Strickland, 1973). **Family-level:** child-parent relationship from the perspective of the child was also assessed	Cross-sectional. Stepwise multiple regression with manifest aggression as the outcome variable	Higher levels of state and trait anxiety were associated with manifest aggression and less internally focused locus of control. An association between children's aggression and poor child-father relationship was also reported
Willcutt et al. (2007)[95]	Longitudinal study of reading disability and attention-deficit/hyperactivity disorder: Implications for education	To investigate the long term academic and psycho-social outcomes of children with reading difficulties (RD) (with and without ADHD)	A sample of children (and their parents) with RD only (n= 71), co-morbid RD/ADHD (n= 51) and ADHD only (n=66) or neither disorder (n=118) Age at initial testing approx. 10-11 years and at the five-year	RD determined by the researchers based on scores on standardised tests of word reading	USA. The children were recruited from an existing 5-year longitudinal twin study	The researchers developed a separate anxiety, depression and externalising symptoms composite score per child based on clinical interview (parents) and scores on standardised self-report measures such as the Children's Depression Inventory (Kovacs, 1988); the Youth Self-report of the Child Behaviour CheckList (CBCL; Achenbach & Rescorla, 2001); and the Children's Manifest Anxiety Scale; Reynolds & Richmond, 1978)	**Individual-level:** academic performance, measured by a composite score combining grade point averages and CBCL parent and self-ratings. **Community-level:** parents and children reported on their social functioning/social difficulties with survey items and the CBCL and YSR-	Longitudinal design. Regression analysis	Reading difficulties were associated with more academic difficulties, depression and adolescent-onset conduct disorder. Co-morbidity of reading difficulties with ADHD increased risk for academic difficulties and social difficulties

Author (Date)	Title	Aim of study	Population	Learning/Reading Difficulties	Context	Mental Health Concept	Third factor of interest	Methodology	Key findings
			follow-up approximately 15-16.3 years				CBCL social problems sub-scale. The survey also contained items to measure children's involvement in extra-curricular activities as an indicator of social engagement		
Willcutt et al. (2013)[96]	Co-morbidity between reading disability and math disability: Concurrent psychopathology, functional impairment, and neuropsychological functioning	To better understand the aetiology of the co-morbidity of mathematics and reading disability	The sample consisted of children and adolescents aged 8-15 years: a group with reading disability (RD group n = 241); Mathematics disability (MD group, n = 183); co-morbid RD and MD (n = 188) and a control group with no known learning disability (n = 411)	RD was defined by a cut-off score 1.25 standard deviations below the estimated population mean (approximately 10th percentile) on an age-adjusted measure of word reading ability derived from the Peabody Individual Achievement Test (PIAT)	USA. The participants were recruited from an existing longitudinal twin study.	Symptoms of internalising/externalising disorders were measured by parent report with the Diagnostic Interview for Children and Adolescents (Reich, Wellner &Herjanic, 1997) and by parent and teacher report with the relevant versions of the Child Behaviour CheckList (CBCL; Achenbach & Rescorla, 1991); and the Disruptive Behaviour Rating Scale (DBRS; Barkley & Murphy, 1998)	**Individual-level:** The child survey measured children's perceptions of their academic achievement**.** Parents reported on their child's global functioning with the Child Global Assessment Scale (Setterber et al., 1992); and parents and teachers reported on children's academic functioning with the relevant section of the DBRS. **Community-level:** Children were asked about the extent to which they liked school and children's social problems were reported on by parents and teachers with the CBCL social problems sub-scale; a rating of peer-liking from teacher's	Cross-sectional. Group comparison design	Higher levels of externalising symptoms appeared to be explained by ADHD in this sample, whereas reading difficulties were associated with higher levels of internalising symptoms (depression and generalized anxiety). Co-morbidity appeared to increase risk for social isolation, peer difficulties and poor academic functioning

Author (Date)	Title	Aim of study	Population	Learning/Reading Difficulties	Context	Mental Health Concept	Third factor of interest	Methodology	Key findings
							perspectives; and the Social Isolation scale from the Colorado Learning Difficulties Questionnaire (CLCQ; Wilcutt et al., 2011)		
Zach et al. (2016)[97]	Academic achievements, behavioral problems, and loneliness as predictors of social skills among students with and without learning disorders	To investigate whether academic achievement, learning difficulties, behaviour problems, and loneliness explain variance in students' social skills	Participants were 733 children and adolescents (6-13 years, 359 boys and 374 girls, 91 with learning difficulties (46 boys, 45 girls), 642 without learning difficulties	All in the LD group had a previous diagnosis based on DSM-4 criteria, sub-type of LD was not described	Israel. Children were all in mainstream school settings	Children's "behaviour problems" were assessed by teacher report with the problem behaviour sub-scale of the Social Skills Rating System (SSRS; Gresham & Elliot, 1990). This sub-scale measures internalising/externalising symptoms and hyperactivity	**Individual-level:** Teachers reported on children's social skills and academic competence with the relevant sub-scales of the SSRT. **Community-level**: Children self-reported on their loneliness with Asher and Wheeler's adapted version of their 24-item self-report measure (Asher, Hymel, and Renshaw, 1984).	Cross-sectional. Regression analysis	LD status did not explain variance in social skills, but academic achievement and behaviour problems did. Loneliness explained social skills variance amongst boys only. This finding suggests an association between social skills and internalising/externalising symptoms
Zuppardo et al., (2021)[98]	More than words: Anxiety, self-esteem and behavioral problems in children and adolescents with dyslexia	To define clinical psycho-affective and behavioural profiles of dyslexia	Children and adolescents (aged 8-18 years) participated including those with dyslexia and a control group without any known learning difficulty	Children and adolescents in the dyslexia group met DSM-5 diagnostic criteria	Italy. All participants were native Italian speakers and recruited with the assistance of a Pediatric Neuropsychiatry service	Children's anxiety was measured by self-report with the Multi-dimensional Anxiety Scale for Children (MASC; March et al., 1997) and by parent report with the Child Behaviour Checklist (CBCL; Achenbach, 1991)	**Individual-level**: children's self-esteem was measured by self-report with Bracken's Multi-dimensional Self-esteem Assessment Test (TMA; Bracken, 1993)	Cross-sectional. A MANCOVA was conducted to compare the groups on the psychosocial variables. A separate analysis looked at group differences related to age (children vs adolescents)	Children and adolescents with dyslexia had lower levels of self-esteem in academic and social situations, higher levels of anxiety and more behavioural problems than their peers without learning difficulties. Compared to adolescents with dyslexia, children with dyslexia appeared to experience more social anxiety, separation anxiety and behavioural problems

References

1. Abarrate, M. Behavior problems and social competence in attention deficit hyperactivity disorder children with and without a learning disorder. Ph.D., United States International University, Ann Arbor, 1993.
2. Aitken, M.; Martinussen, R.; Childs, R.; Tannock, R. Profiles of co-occurring difficulties identified through school-based screening. *Journal of Attention Disorders* **2020**, *24*, 1355-1365, doi:10.1177/1087054716684377.
3. Alexander-Passe, N. How dyslexic teenagers cope: An investigation of self-esteem, coping and depression. *Dyslexia: An International Journal of Research and Practice* **2006**, *12*, 256-275, doi:http://dx.doi.org/10.1002/dys.318.
4. Al-Yagon, M. Comorbid LD and ADHD in childhood: Socioemotional and behavioural adjustment and parents' positive and negative affect. *European Journal of Special Needs Education* **2009**, *24*, 371-391, doi:http://dx.doi.org/10.1080/08856250903223054.
5. Al-Yagon, M. Child-mother and child-father attachment security: Links to internalizing adjustment among children with learning disabilities. *Child Psychiatry & Human Development* **2014**, *45*, 119-131, doi:10.1007/s10578-013-0383-9.
6. Al-Yagon, M. Perceived close relationships with parents, teachers, and peers: Predictors of social, emotional, and behavioral features in adolescents with LD or comorbid LD and ADHD. *Journal of Learning Disabilities* **2016**, *49*, 597-615, doi:10.1177/0022219415620569.
7. Antshel, K.M.; Guy-Ronald, J. Maternal stress in Nonverbal Learning Disorder: A comparison with Reading Disorder. *Journal of Learning Disabilities* **2006**, *39*, 194-205, doi:http://dx.doi.org/10.1177/00222194060390030101.
8. Baumeister, A.L.; Storch, E.A.; Geffken, G.R. Peer victimization in children with learning disabilities. *Child & Adolescent Social Work Journal* **2008**, *25*, 11-23.
9. Benassi, E.; Camia, M.; Giovagnoli, S.; Scorza, M. Impaired school well-being in children with Specific Learning Disorder and its relationship to psychopathological symptoms. *European Journal of Special Needs Education* **2020**, doi:10.1080/08856257.2020.1842975.
10. Blicher, S.; Feingold, L.; Shany, M. The role of trait anxiety and preoccupation with reading disabilities of children and their mothers in predicting children's reading comprehension. *Journal of Learning Disabilities* **2017**, *50*, 309-321, doi:http://dx.doi.org/10.1177/0022219415624101.
11. Boetsch, E.A.; Green, P.A.; Pennington, B.F. Psychosocial correlates of dyslexia across the life span. *Development and Psychopathology* **1996**, *8*, 539-562, doi:http://dx.doi.org/10.1017/S0954579400007264.
12. Bonifacci, P.; Storti, M.; Tobia, V.; Suardi, A. Specific Learning Disorders: A look inside children's and parents' psychological well-being and relationships. *Journal of Learning Disabilities* **2016**, *49*, 532-545, doi:https://dx.doi.org/10.1177/0022219414566681.
13. Bonifacci, P.; Marra, V.; Desideri, L.; Tobia, V.; Baiocco, R.; Ottaviani, C. Rumination and emotional profile in children with Specific Learning Disorders and their parents. *International Journal of Environmental Research and Public Health* **2020**, *17*, 389, doi:http://dx.doi.org/10.3390/ijerph17020389.
14. Boyes, M.E.; Leitão, S.; Claessen, M.; Badcock, N.A.; Nayton, M. Correlates of externalising and internalising problems in children with dyslexia: An analysis of data from clinical casefiles. *Australian Psychologist* **2020**, *55*, 62-72, doi:10.1111/ap.12409.
15. Camilleri, S.; Chetcuti, D.; Falzon, R. "They labeled me ignorant": Narratives of Maltese youth with dyslexia on national examinations. *Sage Open* **2019**, *9*, 1-18, doi:http://dx.doi.org/10.1177/2158244019855674.
16. Capozzi, F.; Casini, M.P.; Romani, M.; De Gennaro, L.; Nicolais, G.; Solano, L. Psychiatric comorbidity in learning disorder: Analysis of family variables. *Child Psychiatry and Human Development* **2008**, *39*, 101-110, doi:http://dx.doi.org/10.1007/s10578-007-0074-5.

17. Carroll, J.M.; Maughan, B.; Goodman, R.; Meltzer, H. Literacy difficulties and psychiatric disorders: Evidence for comorbidity. *Journal of Child Psychology and Psychiatry* **2005**, *46*, 524-532, doi:http://dx.doi.org/10.1111/j.1469-7610.2004.00366.x.
18. Casey, R.; Levy, S.E.; Brown, K.; Brooks-Gunn, J. Impaired emotional health in children with mild reading disability. *Journal of Developmental and Behavioral Pediatrics* **1992**, *13*, 256-260, doi:10.1097/00004703-199208000-00003.
19. Cen, S.; Aytac, B. Ecocultural perspective in learning disability: Family support resources, values, child problem behaviors. *Learning Disability Quarterly* **2017**, *40*, 114-127, doi:10.1177/0731948716683516.
20. Chiappedi, M.; Baschenis, I.M. Specific learning disorders and anxiety: A matter of school experience? *Minerva Pediatr.* **2016**, *68*, 51-55.
21. Claessen, M.; Dzidic, P.; Boyes, M.; Badcock, N.; Nayton, M.; Leitao, S. Educators' perceptions of the impact of reading difficulties for young people. *Australian Journal of Learning Difficulties* **2020**, *25*, 51-64, doi:http://dx.doi.org/10.1080/19404158.2020.1734952.
22. Dahle, A.E.; Knivsberg, A.M.; Andreassen, A.B. Coexisting problem behaviour in severe dyslexia. *Journal of Research in Special Educational Needs* **2011**, *11*, 162-170, doi:10.1111/j.1471-3802.2010.01190.x.
23. Dahle, A.E.; Knivsberg, A.M. Internalizing, externalizing and attention problems in dyslexia. *Scandinavian Journal of Disability Research* **2014**, *16*, 179-193, doi:10.1080/15017419.2013.781953.
24. D'Amico, A.; Guastaferro, T. Emotional and meta-emotional intelligence as predictors of adjustment problems in students with Specific Learning Disorders. *International Journal of Emotional Education* **2017**, *9*, 17-30.
25. DeBrew, J. Who will help my son?: A family's journey with dyslexia. *Journal of Psychosocial Nursing & Mental Health Services* **2017**, *55*, 27-30, doi:http://dx.doi.org/10.3928/02793695-20170718-04.
26. de Lima, R.F.; Salgado-Azoni, C.A.; Dell'Agli, B.A.V.; Baptista, M.N.; Ciasca, S.M. Behavior problems and depressive symptoms in developmental dyslexia: Risk assessment in Brazilian students. *Clinical Neuropsychiatry: Journal of Treatment Evaluation* **2020**, *17*, 141-148.
27. Eissa, M. Behavioral and emotional problems associated with dyslexia in adolescence. *Current Psychiatry* **2010**, *17(1)*, 17-25.
28. Fussell, J.J.; Macias, M.M.; Saylor, C.F. Social skills and behavior problems in children with disabilities with and without siblings. *Child Psychiatry and Human Development* **2005**, *36*, 227-241, doi:10.1007/s10578-005-4185-6.
29. Gadeyne, E.; Ghesquière, P.; Onghena, P. Psychosocial functioning of young children with learning problems. *Journal of Child Psychology & Psychiatry* **2004**, *45*, 510-521, doi:10.1111/j.1469-7610.2004.00241.x.
30. Gallegos, J.; Langley, A.; Villegas, D. Anxiety, depression, and coping skills among Mexican school children: A comparison of students with and without learning disabilities. *Learning Disability Quarterly* **2012**, *35*, 54-61, doi:10.1177/0731948711428772.
31. Giovagnoli, S.; Mandolesi, L.; Magri, S.; Gualtieri, L.; Fabbri, D.; Tossani, E.; Benassi, M. Internalizing symptoms in developmental dyslexia: A comparison between primary and secondary school. *Frontiers in Psychology* **2020**, *11*, 461, doi:https://dx.doi.org/10.3389/fpsyg.2020.00461.
32. Goldston, D.B.; Walsh, A.; Arnold, E.M.; Reboussin, B.; Daniel, S.S.; Erkanli, A.; Nutter, D.; Hickman, E.; Palmes, G.; Snider, E.; et al. Reading problems, psychiatric disorders, and functional impairment from mid- to late adolescence. *Journal of the American Academy of Child & Adolescent Psychiatry* **2007**, *46*, 25-32, doi:http://dx.doi.org/10.1097/01.chi.0000242241.77302.f4.
33. Granot, D. Socioemotional and behavioural adaptation of students with disabilities: The significance of teacher–student attachment-like relationships. *Emot. Behav. Difficulties* **2016**, *21*, 416-432, doi:10.1080/13632752.2016.1235324.
34. Haager, D.; Vaughn, S. Parent, teacher, peer, and self-reports of the social competence of students with learning disabilities. *Journal of Learning Disabilities* **1995**, *28*, 205-231, doi:10.1177/002221949502800403.

35. Heiervang, E.; Stevenson, J.; Lund, A.; Hugdahl, K. Behaviour problems in children with dyslexia. *Nordic Journal of Psychiatry* **2001**, *55*, 251-256, doi:10.1080/080394801681019101.
36. Heying, K.R. Social competence and behavioural problems in reading-disabled boys with and without attention deficit disorder Ph.D., United States International University, Ann Arbor, 1987.
37. Hirsh, R.A. Emotional responses to the reading difficulties experienced by young children enrolled in Title I: A qualitative study of students', teachers', and families' perspectives. PhD, Indiana University of Pennsylvania, Ann Arbor, 2014.
38. Horbach, J.; Mayer, A.; Scharke, W.; Heim, S.; Günther, T. Development of behavior problems in children with and without Specific Learning Disorders in reading and spelling from kindergarten to fifth grade. *Scientific Studies of Reading* **2020**, *24*, 57-71, doi:http://dx.doi.org/10.1080/10888438.2019.1641504.
39. Hossain, B.; Bent, S.; Hendren, R. The association between anxiety and academic performance in children with reading disorder: A longitudinal cohort study. *Dyslexia* **2021**, *27(3)*, 342-354, doi:http://dx.doi.org/10.1002/dys.1680.
40. Hossain, B.; Chen, Y.; Bent, S.; Parenteau, C.; Widjaja, F.; Haft, S.L.; Hoeft, F.; Hendren, R.L. The role of grit and resilience in children with reading disorder: A longitudinal cohort study. *Annals of Dyslexia* **2022**, *72*, 1-27, doi:https://dx.doi.org/10.1007/s11881-021-00238-w.
41. Hughes, W.; Dawson, R.O.N. Memories of school: Adult dyslexics recall their school days. *Support for learning* **1995**, *10*, 181-184, doi:10.1111/j.1467-9604.1995.tb00037.x.
42. Ihbour, S.; Anarghou, H.; Boulhana, A.; Najimi, M.; Chigr, F. Mental health among students with neurodevelopment disorders: Case of dyslexic children and adolescents. *Dementia & Neuropsychologia* **2021**, *15*, 533-540, doi:https://dx.doi.org/10.1590/1980-57642021dn15-040014.
43. Ilardi, M. Maternal mentalization and child psychosocial adaptation for children with learning and behavioral disorders. Ph.D., City University of New York, Ann Arbor, 2010.
44. Ingesson, S.G. Growing up with dyslexia: Interviews with teenagers and young adults. *School Psychology International* **2007**, *28*, 574-591, doi:http://dx.doi.org/10.1177/0143034307085659.
45. Iyanda, A.E. Bullying victimization of children with mental, emotional, and developmental or behavioral (MEDB) disorders in the United States. *J. Child Adolesc. Trauma* **2021**, doi:http://dx.doi.org/10.1007/s40653-021-00368-8.
46. Jordan, J.-A.; Dyer, K. Psychological well-being trajectories of individuals with dyslexia aged 3-11 years. *Dyslexia* **2017**, *23*, 161-180, doi:http://dx.doi.org/10.1002/dys.1555.
47. Kempe, C.; Gustafson, S.; Samuelsson, S. A longitudinal study of early reading difficulties and subsequent problem behaviors. *Scandinavian Journal of Psychology* **2011**, *52*, 242-250, doi:10.1111/j.1467-9450.2011.00870.x.
48. Konstantareas, M.M.; Homatidis, S. Parental perception of learning-disabled children's adjustment problems and related stress. *Journal of Abnormal Child Psychology* **1989**, *17*, 177-186, doi:10.1007/BF00913792.
49. Kopelman-Rubin, D.; Siegel, A.; Weiss, N.; Kats-Gold, I. The relationship between emotion regulation, school belonging, and psychosocial difficulties among adolescents with Specific Learning Disorder. *Children & Schools* **2020**, *42*, 216-224, doi:http://dx.doi.org/10.1093/cs/cdaa022.
50. La Greca, A.M.; Stone, W.L. LD status and achievement: confounding variables in the study of children's social status, self-esteem, and behavioral functioning. *Journal of Learning Disabilities* **1990**, *23*, 483-490, doi:10.1177/002221949002300806.
51. Lawrence, B.C.; Harrison, G.L.; Milford, T.M. Effects of positive parenting on mental health in adolescents with learning disabilities. *Learning Disabilities (Weston, Mass.)* **2019**, *17*, 223.
52. Lawrence, B.C. Bridging secondary survey data with in-depth case studies to advance understandings of youth learning and mental health concerns. *International Journal of Multiple Research Approaches* **2020**, *12*, 166-184, doi:10.29034/ijmra.v12n2a1.

53. Learned, J.E. The Behavior Kids": examining the conflation of youth reading difficulty and behavior problem positioning among school institutional contexts. *American Educational Research Journal* **2016**, *53*, 1271-1309, doi:http://dx.doi.org/10.3102/0002831216667545.
54. Leitão, S.; Dzidic, P.; Claessen, M.; Gordon, J.; Howard, K.; Nayton, M.; Boyes, M.E. Exploring the impact of living with dyslexia: The perspectives of children and their parents. *International Journal of Speech-Language Pathology* **2017**, *19*, 322-334, doi:10.1080/17549507.2017.1309068.
55. Lin, Y.C.; Morgan, P.L.; Hillemeier, M.; Cook, M.; Maczuga, S.; Farkas, G. Reading, mathematics, and behavioral difficulties interrelate: Evidence from a cross-lagged panel design and population-based sample of US upper elementary students. *Behavioral Disorders* **2013**, *38*, 212-227, doi:10.1177/019874291303800404.
56. Lindeblad, E.; Svensson, I.; Gustafson, S. Self-concepts and psychological well-being assessed by Beck Youth Inventory among pupils with reading difficulties. *Reading Psychology* **2016**, *37*, 449-469, doi:http://dx.doi.org/10.1080/02702711.2015.1060092.
57. Lytton, H. Some psychological and sociological characteristics of "good" and "poor achievers" (boys) in remedial reading groups: Clinical case studies. *Human Development* **1968**, *11*, 260-276, doi:http://dx.doi.org/10.1159/000270611.
58. Mahdavi, S. The Influence of early parent involvement and depression on academic achievement, psychosocial behaviors, and motivation in children with learning disabilities across elementary school. PhD, Michigan State University, Ann Arbor, 2017.
59. Martinez, R.S. A comparison of learning disability subtypes in middle school: Self-concept, perceived social support, and emotional functioning. Ph.D., The University of Texas at Austin, Ann Arbor, 2002.
60. Matteucci, M.C.; Scalone, L.; Tomasetto, C.; Selleri, P.; Cavrini, G. Health-related quality of life and psychological wellbeing of children with Specific Learning Disorders and their mothers. *Research in Developmental Disabilities* **2019**, *87*, 43-53, doi:http://dx.doi.org/10.1016/j.ridd.2019.02.003.
61. Maughan, B.; Rowe, R.; Loeber, R.; Stouthamer-loeber, M. Reading problems and depressed mood. *Journal of Abnormal Child Psychology* **2003**, *31*, 219-229, doi:http://dx.doi.org/10.1023/A:1022534527021.
62. McArthur, G.; Badcock, N.; Castles, A.; Robidoux, S. Tracking the relations between children's reading and emotional health across time: Evidence from four large longitudinal studies. *Reading Research Quarterly* **2022**, *57*, 555-585, doi:https://doi.org/10.1002/rrq.426.
63. Mercer, K.L. Relations of self-efficacy to symptoms of depression and anxiety in adolescents with learning disabilities. Ph.D., The University of British Columbia (Canada), Ann Arbor, 2004.
64. Michaels, C.R.; Lewandowski, L.J. Psychological adjustment and family functioning of boys with learning disabilities. *Journal of Learning Disabilities* **1990**, *23*, 446-450, doi:10.1177/002221949002300709.
65. Morgan, P.L.; Farkas, G.; Tufis, P.A.; Sperling, R.A. Are reading and behavior problems risk factors for each other? *Journal of Learning Disabilities* **2008**, *41*, 417-436, doi:http://dx.doi.org/10.1177/0022219408321123.
66. Morgan, P.L.; Farkas, G.; Wu, Q. Do poor readers feel angry, sad, and unpopular? *Scientific Studies of Reading* **2012**, *16*, 360, doi:http://dx.doi.org/10.1080/10888438.2011.570397.
67. Newbury, J.; Justice, L.M.; Jiang, H.H.; Schmitt, M.B. Cognitive, noncognitive, and home environment correlates of reading difficulties in primary-grade students with language impairment. *Journal of Speech, Language & Hearing Research* **2020**, *63*, 1933-1946, doi:10.1044/2020_jslhr-19-00363.
68. Novita, S. Secondary symptoms of dyslexia: A comparison of self-esteem and anxiety profiles of children with and without dyslexia. *European Journal of Special Needs Education* **2016**, *31*, 279-288, doi:http://dx.doi.org/10.1080/08856257.2015.1125694.

69. Novita, S.; Uyun, Q.; Witruk, E.; Siregar, J.R. Children with dyslexia in different cultures: Investigation of anxiety and coping strategies of children with dyslexia in Indonesia and Germany. *Annals of Dyslexia* **2019**, *69*, 204-218, doi:10.1007/s11881-019-00179-5.
70. Operto, F.F.; Smirni, D.; Scuoppo, C.; Padovano, C.; Vivenzio, V.; Quatrosi, G.; Carotenuto, M.; Precenzano, F.; Pastorino, G.M.G. Neuropsychological profile, emotional/behavioral problems, and parental stress in children with neurodevelopmental disorders. *Brain Sciences* **2021**, *11*, doi:10.3390/brainsci11050584.
71. Palti, G. A study of the socio-emotional aspects of educationally resilient dyslexic pupils. Ph.D., University of Bristol (United Kingdom), Ann Arbor, 1998.
72. Panicker, A.S.; Chelliah, A. Resilience and stress in children and adolescents with Specific Learning Disability. *Journal of the Canadian Academy of Child & Adolescent Psychiatry* **2016**, *25*, 17-23.
73. Parhiala, P.; Torppa, M.; Eklund, K.; Aro, T.; Poikkeus, A.M.; Heikkila, R.; Ahonen, T. Psychosocial functioning of children with and without dyslexia: A follow-up study from ages four to nine. *Dyslexia* **2015**, *21*, 197-211, doi:http://dx.doi.org/10.1002/dys.1486.
74. Pih, G.F. A comparison of normal and disabled readers in elementary school on intellectual, self-esteem, and anxiety factors Ph.D., University of Georgia, Ann Arbor, 1984.
75. Russell, G.; Ford, T.; Ryder, D.; Norwich, B. Behavioural difficulties that co-occur with specific word reading difficulties: A UK population-based cohort study. *Dyslexia* **2015**, *21*, 123-141, doi:http://dx.doi.org/10.1002/dys.1496.
76. Sahin, B.; Karabekiroglu, K.; Aydin, M.; Cobanoglu, C.; Bozkurt, A.; Usta, M.B. The relationship of clinical symptoms with social cognition in children diagnosed with attention deficit hyperactivity disorder, specific learning disorder or autism spectrum disorder. *Psychiatry Investigation* **2018**, *15*, 1144-1153, doi:http://dx.doi.org/10.30773/PI.2018.10.01.
77. Sainio, P.J.; Eklund, K.M.; Ahonen, T.P.S.; Kiuru, N.H. The role of learning difficulties in adolescents' academic emotions and academic achievement. *Journal of Learning Disabilities* **2019**, *52*, 287-298, doi:http://dx.doi.org/10.1177/0022219419841567.
78. Singer, E. The strategies adopted by Dutch children with dyslexia to maintain their self-esteem when teased at school. *Journal of Learning Disabilities* **2005**, *38*, 411-423, doi:http://dx.doi.org/10.1177/00222194050380050401.
79. Singer, E. Coping with academic failure, a study of Dutch children with dyslexia. *Dyslexia* **2007**, *14*, 314-333, doi:10.1002/dys.352.
80. Smart, D.; Youssef, G.J.; Sanson, A.; Prior, M.; Toumbourou, J.W.; Olsson, C.A. Social mediators of relationships between childhood reading difficulties, behaviour problems, and secondary school noncompletion. *Australian Journal of Psychology* **2019**, *71*, 171-182, doi:http://dx.doi.org/10.1111/ajpy.12220.
81. Snowling, M.J.; Muter, V.; Carroll, J. Children at family risk of dyslexia: A follow-up in early adolescence. *Journal of Child Psychology and Psychiatry* **2007**, *48*, 609-618, doi:http://dx.doi.org/10.1111/j.1469-7610.2006.01725.x.
82. Sorrenti, L.; Spadaro, L.; Mafodda, A.V.; Scopelliti, G.; Orecchio, S.; Filippello, P. The predicting role of school learned helplessness in internalizing and externalizing problems: An exploratory study in students with Specific Learning Disorder. *Mediterr. J. Clin. Psychol.* **2019**, *7*, 1-13, doi:10.6092/2282-1619/2019.7.2035.
83. Speers, J. An auto/biographical investigation exploring the life-stories of adults with dyslexia. PhD thesis, Canterbury Christ Church University (United Kingdom), Ann Arbor, 2019.
84. Stanley, P.D.; Dai, Y.; Nolan, R.F. Differences in depression and self-esteem reported by learning disabled and behavior disordered middle school students. *Journal of Adolescence* **1997**, *20*, 219-222, doi:10.1006/jado.1996.0079.
85. Sukovieff, A.; Kruk, R.S. Reading difficulty and socio-emotional adjustment: Internalizing patterns depend on age of identification. *Cogent Educ.* **2021**, *8*, doi:10.1080/2331186X.2021.1910162.

86. Terras, M.M.; Thompson, L.C.; Minnis, H. Dyslexia and psycho-social functioning: An exploratory study of the role of self-esteem and understanding. *Dyslexia* **2009**, *15*, 304-327, doi:10.1002/dys.386.
87. Thompson Jr, R.J.; Lampron, L.B.; Johnson, D.F.; Eckstein, T.L. Behavior problems in children with the presenting problem of poor school performance. *J. Pediatr. Psychol.* **1990**, *15*, 3-20.
88. Tistan, P.A. Learning disabilities and depression in children: The development of self-concept and achievement. Ph.D., Seton Hall University, School of Education, Ann Arbor, 1993.
89. Törő, K.T.; Miklósi, M.; Horanyi, E.; Kovács, G.P.; Balázs, J. Reading disability spectrum: Early and late recognition, subthreshold, and full comorbidity. *Journal of Learning Disabilities* **2018**, *51*, 158-167, doi:10.1177/0022219417704169.
90. Tsovili, T.D. The relationship between language teachers' attitudes and the state-trait anxiety of adolescents with dyslexia. *Journal of Research in Reading* **2004**, *27*, 69-86, doi:http://dx.doi.org/10.1111/j.1467-9817.2004.00215.x.
91. Turunen, T.; Kiuru, N.; Poskiparta, E.; Niemi, P.; Nurmi, J.E. Word reading skills and externalizing and internalizing problems from grade 1 to grade 2—Developmental trajectories and bullying involvement in grade 3. *Scientific Studies of Reading* **2019**, *23*, 161-177, doi:10.1080/10888438.2018.1497036.
92. Undheim, A.M.; Sund, A.M. Psychosocial factors and reading difficulties: Students with reading difficulties drawn from a representative population sample. *Scandinavian Journal of Psychology* **2008**, *49*, 377-384, doi:10.1111/j.1467-9450.2008.00661.x.
93. Undheim, A.M.; Wichstrøm, L.; Sund, A.M. Emotional and behavioral problems among school adolescents with and without reading difficulties as measured by the Youth Self-report: A one-year follow-up study. *Scandinavian Journal of Educational Research* **2011**, *55*, 291-305, doi:10.1080/00313831.2011.576879.
94. Valenti, F.R. Manifest aggression, state/trait anxiety, locus of control, perceived parental attention, and reading level among learning disabled adolescent males, PhD thesis, Boston University, Ann Arbor, 1986.
95. Willcutt, E.G.; Betjemann, R.S.; Pennington, B.F.; Olson, R.K.; DeFries, J.C.; Wadsworth, S.J. Longitudinal study of reading disability and attention-deficit/hyperactivity disorder: Implications for education. *Mind, Brain, and Education* **2007**, *1*, 181-192, doi:http://dx.doi.org/10.1111/j.1751-228X.2007.00019.x.
96. Willcutt, E.G.; Petrill, S.A.; Wu, S.; Boada, R.; DeFries, J.C.; Olson, R.K.; Pennington, B.F. Comorbidity between reading disability and math disability: Concurrent psychopathology, functional impairment, and neuropsychological functioning. *Journal of Learning Disabilities* **2013**, *46*, 500-516, doi:10.1177/0022219413477476.
97. Zach, S.; Yazdi-Ugav, O.; Zeev, A. Academic achievements, behavioral problems, and loneliness as predictors of social skills among students with and without learning disorders. *School Psychology International* **2016**, *37*, 378-396, doi:10.1177/0143034316649231.
98. Zuppardo, L.; Serrano, F.; Pirrone, C.; Rodriguez-Fuentes, A. More than words: Anxiety, self-esteem and behavioral problems in children and adolescents with dyslexia. *Learning Disability Quarterly* **2021**, doi:10.1177/07319487211041103.
